# Predicted impacts of heterogeneous chemical pathways on particulate sulfur over Fairbanks (Alaska), the Northern Hemisphere, and the Contiguous United States

**DOI:** 10.5194/acp-25-3287-2025

**Published:** 2025-03-18

**Authors:** Sara L. Farrell, Havala O. T. Pye, Robert Gilliam, George Pouliot, Deanna Huff, Golam Sarwar, William Vizuete, Nicole Briggs, Fengkui Duan, Tao Ma, Shuping Zhang, Kathleen Fahey

**Affiliations:** 1 Department of Environmental Sciences and Engineering, The University of North Carolina at Chapel Hill, Chapel Hill, NC 27516, USA; 2 Office of Research and Development, U.S. Environmental Protection Agency, Research Triangle Park, Durham, NC 27709, USA; 3 Oak Ridge Institute for Science and Education, U.S. Environmental Protection Agency, Research Triangle Park, Durham, NC 27709, USA; 4 Alaska Department of Environmental Conservation, P.O. Box 111800, Juneau, AK 99811-1800, USA; 5 Laboratory Services and Applied Science Division at USEPA, Region 10, Seattle, WA 98101, USA; 6 State Key Joint Laboratory of Environment Simulation and Pollution Control, School of Environment, Tsinghua University, Beijing 100084, China; 7 Guangdong-Hong Kong-Macao Joint Laboratory for Contaminants Exposure and Health, Guangdong Key Laboratory of Environmental Catalysis and Health Risk Control, School of Environmental Science and Engineering, Institute of Environmental Health and Pollution Control, Guangdong University of Technology, Guangzhou 510006, China

## Abstract

A portion of Alaska’s Fairbanks North Star Borough was designated as nonattainment for the 2006 24 h fine particulate matter 2.5 μm or less in diameter (PM_2.5_) National Ambient Air Quality Standards (NAAQS) in 2009. PM_2.5_ NAAQS exceedances in Fairbanks mainly occur during dark and cold winters, when temperature inversions form and trap high emissions at the surface. Sulfate (${\text{SO}}_{4}^{2-}$), often the second-largest contributor to PM_2.5_ mass during these wintertime PM episodes, is underpredicted by atmospheric chemical transport models (CTMs). Most CTMs account for primary ${\text{SO}}_{4}^{2-}$ and secondary ${\text{SO}}_{4}^{2-}$ formed via gas-phase oxidation of sulfur dioxide (${\text{SO}}_{2}$) and in-cloud aqueous oxidation of dissolved S(IV). Dissolution and reaction of ${\text{SO}}_{2}$ in aqueous aerosols are generally not included in CTMs but can be represented as heterogeneous reactive uptake and may help better represent the high ${\text{SO}}_{4}^{2-}$ concentrations observed during Fairbanks winters. In addition, hydroxymethanesulfonate (HMS), a particulate sulfur species sometimes misidentified as ${\text{SO}}_{4}^{2-}$, is known to form during Fairbanks winters. Heterogeneous formation of ${\text{SO}}_{4}^{2-}$ and HMS in aerosol liquid water (ALW) was implemented in the Community Multiscale Air Quality (CMAQ) modeling system. CMAQ simulations were performed for wintertime PM episodes in Fairbanks (2008) as well as over the Northern Hemisphere and Contiguous United States (CONUS) for 2015–2016. The added heterogeneous sulfur chemistry reduced model mean sulfate bias by ~0.6 μg m^−3^ during a cold winter PM episode in Fairbanks, AK. Improvements in model performance are also seen in Beijing during wintertime haze events (reducing model mean sulfate bias by ~2.9 μgS m^−3^). This additional sulfur chemistry also improves modeled summertime ${\text{SO}}_{4}^{2-}$ bias in the southeastern US, with implications for future modeling of biogenic organosulfates.

## 1 Introduction

Radiative forcing and climate effects attributed to fine particulate matter 2.5 μm or less in diameter (PM_2.5_) remain among the most uncertain in climate change assessments (IPCC, 2013). Acute and long-term exposure to PM_2.5_ has been associated with negative health outcomes including but not limited to acute myocardial infarction, stroke, and respiratory complications (Chen et al., 2018; Hayes et al., 2020; Silva et al., 2016; USEPA, 2019; Yang et al., 2021; Yitshak-Sade et al., 2018). Some of the most extreme PM_2.5_ episodes in history have occurred when inverted vertical temperature profiles cause stable atmospheric conditions that limit pollution dilution (Holzworth, 1972; Malek et al., 2006; Scott, 1953; Wallace and Kanaroglou, 2009). Near-surface temperature inversions (with a minimum modeled planetary boundary layer height of 1.7 m) are characteristic of Fairbanks and North Pole (Alaska) winters and are associated with degraded air quality in this region (Malingowski et al., 2014; Mayfield and Fochesatto, 2013). Wintertime PM_2.5_ episodes have impacted human health in these cities (McLaughlin and Castrodale, 2010), with this region exceeding the National Ambient Air Quality Standards (NAAQS) for PM_2.5_ since 2009, when portions of the Fairbanks North Star Borough were designated as nonattainment for the 2006 24 h PM_2.5_ NAAQS^1^ (ADEC, 2017, 2019).

Sulfate (${\text{SO}}_{4}^{2-}$), often a major component of PM_2.5_ in Fairbanks and North Pole (ADEC, 2017) as well as globally (Snider et al., 2016), can be emitted directly (primary) or formed secondarily via atmospheric oxidation of sulfur dioxide (${\text{SO}}_{2}$). Known secondary ${\text{SO}}_{4}^{2-}$ formation processes include but are not limited to gas-phase oxidation of ${\text{SO}}_{2}$ (Calvert et al., 1978), particle surface oxidation of ${\text{SO}}_{2}$ (Clements et al., 2013; Wang et al., 2021), and in-cloud aqueous-phase oxidation of inorganic sulfur species with oxidation number $4(S(\text{IV})={\text{SO}}_{2}\cdot H_{2}O+{\text{HSO}}_{3}^{-}+{\text{SO}}_{3}^{2-})$ (secondary) (Hoffmann and Calvert, 1985; Ibusuki and Takeuchi, 1987; Lagrange et al., 1994; Lee and Schwartz, 1983a; Maahs, 1983; Maaß et al., 1999; Martin and Good, 1991; McArdle and Hoffmann, 1983). Aside from contributing directly to PM_2.5_ mass, ${\text{SO}}_{4}^{2-}$ can facilitate the formation of other PM_2.5_ species as a reactant (Brüggemann et al., 2020; Huang et al., 2019; Huang et al., 2020; Surratt et al., 2010) by increasing aerosol water uptake (Kim et al., 1994; Nguyen et al., 2014) and by altering aerosol acidity (Li et al., 2022; Pye et al., 2020).

Under heavily polluted haze conditions, such as those common in the North China Plain during the winter, recent studies have suggested that secondary ${\text{SO}}_{4}^{2-}$ may be efficiently produced in aerosol liquid water (ALW) (Cheng et al., 2016; Fan et al., 2020; Liu et al., 2020). Hygroscopic PM_2.5_ (both inorganic and organic) can increase ALW content (Nguyen et al., 2014; Petters and Kreidenweis, 2007; Pye et al., 2017), which can facilitate secondary ${\text{SO}}_{4}^{2-}$ formation (Zhang et al., 2021b), enhancing ${\text{SO}}_{4}^{2-}$ concentrations in a positive feedback loop – which is particularly important during high-relative-humidity haze events (Cheng et al., 2016; Song et al., 2021a; Wang et al., 2016, 2014).

Chemical transport models generally include secondary ${\text{SO}}_{4}^{2-}$ formation via gas-phase oxidation of ${\text{SO}}_{2}$ by OH and in-cloud aqueous-phase oxidation of dissolved S(IV) species by oxidants such as hydrogen peroxide ($H_{2}O_{2}$); ozone ($O_{3}$); peroxyacetic acid (PAA); methyl hydroperoxide (MHP); and/or oxygen ($O_{2}$) catalyzed by transition metal ions (TMIs-$O_{2}$), iron (${\text{Fe}}^{3+}$), and manganese (${\text{Mn}}^{2+}$). Limited to these formation pathways, CTMs have been unable to reproduce the high levels of ${\text{SO}}_{4}^{2-}$ observed during wintertime PM events in Beijing and regions that experience extremely cold winters. This persistent underprediction suggests that CTMs lack ${\text{SO}}_{4}^{2-}$ formation pathways that take place when photochemistry and cloud liquid water are limited (Eckhardt et al., 2015; Gao et al., 2016; Wang et al., 2014). Previous studies have suggested that heterogeneous sulfate formation in deliquesced aerosol particles may account for at least part of this underprediction (Wang et al., 2014; Zheng et al., 2015). Wang et al. (2014) implemented generalized heterogeneous reactive uptake of ${\text{SO}}_{2}$ to form ${\text{SO}}_{4}^{2-}$ (where relative-humidity-dependent uptake coefficients were specified rather than calculated) in the GEOS-Chem global model (Chen et al., 2009), and this led to improved model–observation comparisons for ${\text{SO}}_{4}^{2-}$ during a wintertime haze event over North China (Wang et al., 2014). Similarly, when Zheng et al. (2015) implemented generalized heterogeneous sulfur chemistry in WRF-CMAQ, the ${\text{SO}}_{4}^{2-}$ normalized mean bias decreased from −54.2% to 6.3% for Beijing haze events (Zheng et al., 2015).

The inclusion of hydroxymethanesulfonate (HMS) chemistry in CTMs may also ameliorate negative model bias. General ${\text{SO}}_{4}^{2-}$ PM measurement methods struggle to disentangle ${\text{SO}}_{4}^{2-}$ spectra from those of inorganic S(IV) and HMS (Dovrou et al., 2019). HMS is formed from the aqueousphase reaction of S(IV) with formaldehyde (HCHO) (Boyce and Hoffmann, 1984; Deister et al., 1986; Kok et al., 1986; Kovacs et al., 2005; Olson and Hoffmann, 1986). Recent field, modeling, and experimental studies have highlighted the importance of secondary ${\text{SO}}_{4}^{2-}$ and HMS formation in aqueous aerosols during wintertime haze events (Campbell et al., 2022; Dovrou et al., 2019; Ma et al., 2020; Moch et al., 2020; Song et al., 2019). Moch et al. (2020) suggested up to 25% of measured ${\text{SO}}_{4}^{2-}$ may actually be HMS in heavily polluted regions. Arctic, sub-Arctic, and regions that experience extremely cold and dark winters may favor HMS formation, as colder temperatures increase the solubility of ${\text{SO}}_{2}$ and HCHO, and limited sunlight reduces the photo-oxidation of HCHO (Moch et al., 2020; Pandis and Seinfeld, 1989; Sander, 2015; Song et al., 2019; Staudinger and Roberts, 1996). In the work of Song et al. (2021a), including generalized heterogeneous cloud HMS chemistry in GEOS-Chem reduced model–measurement differences in $\text{HMS}∕{\text{SO}}_{4}^{2-}$ ratios. The inclusion of heterogeneous formation and loss of HMS in deliquesced aerosols increased modeled HMS concentrations, particularly in China (Song et al., 2021b).

Exploring heterogeneous sulfur chemistry in deliquesced aerosols requires retrofitting of multi-phase reactions. Many laboratory studies characterizing rate coefficients and expressions for aqueous-phase ${\text{SO}}_{4}^{2-}$ formation have been performed under dilute conditions, characteristic of cloud droplets. The ionic strength of aerosol particles, however, can be several orders of magnitude higher than that of cloud droplets due to aerosols containing much lower concentrations of water (Mekic and Gligorovski, 2021). Ionic strength has been found to impact the aqueous-phase formation of ${\text{SO}}_{4}^{2-}$, increasing the rate of aqueous-phase kinetics for ${\text{NO}}_{2}$ and $H_{2}O_{2}$ oxidation of S(IV) (Chen et al., 2019; Liu et al., 2020) and inhibiting the aqueous-phase kinetics of TMI-catalyzed $O_{2}$ oxidation (Ibusuki and Takeuchi, 1987; Martin and Good, 1991; Martin and Hill, 1987). Experimental studies have also shown that high ionic strength may increase or decrease (effective) Henry’s law coefficients of reactants compared to pure water (Ali et al., 2014; Chen et al., 2019; Kosak-Channing and Helz, 1983; Lagrange et al., 1994; Liu et al., 2020; Millero et al., 1989; Shao et al., 2019).

In this paper we describe the implementation of heterogenous sulfur chemistry in ALW in the Community Multiscale Air Quality (CMAQ) v5.3.2 modeling system (USEPA, 2020), leading to additional ${\text{SO}}_{4}^{2-}$ and HMS formation. We refer to this chemistry as heterogeneous given the use of the heterogeneous framework (Hanson et al., 1994). Heterogeneous sulfur chemistry pathways implemented include the oxidation of dissolved S(IV) species by $H_{2}O_{2}$, $O_{3}$, PAA, MHP, $\text{TMI-}O_{2}$, and ${\text{NO}}_{2}$ and the in-aerosol aqueous formation of HMS. In addition to heterogeneous chemistry updates, ionic strength effects were added to condensed-phase rate expressions and Henry’s law coefficients of some species. The updated model was applied for several time periods and for different domains and horizonal resolutions. Two historical wintertime PM episodes were simulated for a finely resolved (1.33 km) domain centered over Fairbanks, Alaska; winter and summer periods over the Contiguous United States (CONUS) (12 km) during 2016; and the 2015–2016 winter season over the Northern Hemisphere (108 km) to investigate the impacts of these updates for different chemical regimes, domains, and seasons. Changes to ${\text{SO}}_{4}^{2-}$, HMS, and ${\text{SO}}_{4}^{2-}+\text{HMS}\;({\text{PM}}_{2.5,\text{sulf}})$ predictions were tracked with each update (i.e., for (1) adding heterogeneous sulfur reactions and (2) adding ionic strength effects), and model performance was evaluated with available observations. This study aims to better understand the impacts that heterogeneous sulfur chemistry parameterizations may have on predicted ${\text{PM}}_{2.5,\text{sulf}}$ concentrations and whether the additional chemistry can resolve ${\text{SO}}_{4}^{2-}$ underpredictions in cold and dark conditions.

## 2 Methods

### 2.1 Heterogeneous sulfur chemistry

In this study, reactions that transform ${\text{SO}}_{2}$ to ${\text{SO}}_{4}^{2-}$ and HMS are simulated in both clouds and ALW. In ALW, the production of particulate ${\text{SO}}_{4}^{2-}$ and HMS is parameterized as a set of first-order heterogeneous reactions of ${\text{SO}}_{2}$ (gas) or reactants (e.g., HCHO, $O_{3}$, ${\text{NO}}_{2}$) as follows:

(R1) $$P_{{\text{SO}}_{2},\text{reactant}}\overset{k_{\text{het}}}{\to }{\text{PM}}_{2.5,\text{sulf}},$$

where ${\text{PM}}_{2.5,\text{sulf}}$ refers to both sulfate and HMS, and $k_{\text{het}}$ is the heterogeneous rate constant (Eq. 1), which accounts for gas-to-particle mass transfer processes, aqueous reactive uptake, and sulfur transformations (Jacob, 2000).


(1) $$k_{\text{het}}\left(s^{-1}\right)=\frac{\text{SA}}{\frac{r_{p}}{D_{g}}+\frac{4}{v\gamma }}$$

Here, SA is the aerosol surface area ($m^{2}m^{-3}$), $r_{p}$ is the effective particle radius (m), $D_{g}$ is gas-phase diffusivity of a reactant ($m^{2}\;s^{-1}$), and v is the mean molecular speed of the partitioning gas ($m\;s^{-1}$). γ is the reactive uptake coefficient and is given by the following equation (Hanson et al., 1994; Jacob, 2000; Schwartz, 1986):

(2) $$\gamma ={\left[\frac{1}{\alpha }+\frac{v}{4HRT\sqrt{D_{a}k_{\text{chem}}}}\cdot \frac{1}{\text{coth}(q)-\frac{1}{q}}\right]}^{-1},$$

where α is the mass accommodation coefficient of a species, H is the effective Henry’s law coefficient (M atm^−1^) at temperature T(K), R is the ideal gas constant 0.08206 (L atm mol^−1^ K^−1^), $D_{a}$ is the aqueous-phase diffusivity (assumed here to be 10^−9^ m^2^ s^−1^), $k_{\text{chem}}$ represents the pseudo-first-order condensed-phase rate coefficient (s^−1^) (Table 1), and q is the diffuso-reactive parameter defined as $q=r_{p}\sqrt{\frac{k_{\text{chem}}}{D_{a}}}$ (Schwartz and Freiberg, 1981). The rate expressions in aerosol water for the base configuration (hereafter referred to as “Base_Het”) are the same as those used for cloud chemistry (Appel et al., 2017; Sarwar et al., 2013). Heterogeneous sulfur chemistry was calculated when relative humidity (RH) was greater than or equal to 50% following Shao et al. (2019), assuming that below a 50% RH, the aerosol water content would be too low for heterogeneous sulfur chemistry to take place (Sun et al., 2013). The heterogeneous chemistry is also solved simultaneously with gas-phase chemistry.

### 2.2 Accounting for ionic strength effects and alternative chemical rate expressions

Several studies have investigated the impact of high ionic strength on sulfate oxidation rates originally developed for dilute conditions. Depending on the reaction pathway, rates may be enhanced or diminished with increased ionic strength (Chen et al., 2019; Lagrange et al., 1994; Liu et al., 2020; Shao et al., 2019). Ionic strength is calculated in CMAQ as

(3) $$I=\frac{1}{2}\sum_{i=1}^{n}m_{i}z_{i}^{2},$$

where $m_{i}$ is the solute concentration (M) in aerosol or cloud liquid water, and $z_{i}$ is the charge associated with each modeled ion.

${\text{SO}}_{2}$ can be oxidized in the aqueous phase by $O_{2}$ when catalyzed by TMI (specifically ${\text{Fe}}^{3+}$ and ${\text{Mn}}^{2+}$) with synergies existing when both ${\text{Fe}}^{3+}$ and ${\text{Mn}}^{2+}$ are present (Altwicker and Nass, 1983; Ibusuki and Takeuchi, 1987; Martin and Good, 1991). This reaction pathway has been found to be an important secondary ${\text{SO}}_{4}^{2-}$ formation pathway, especially at low pH and when photochemistry is limited and Fe is more soluble (Cheng et al., 2016; Li et al., 2020; Liu et al., 2022; Song et al., 2021a). Ibusuki and Takeuchi (1987) found that the rate of S(IV) oxidation by the TMI-$O_{2}$ pathway peaked around pH = 4.2, decreased with decreased temperature, and was enhanced by high concentrations of TMI (Ibusuki and Takeuchi, 1987). Martin and Good (1991) investigated the impact of higher S(IV) concentrations on this ${\text{SO}}_{4}^{2-}$ formation pathway and found that higher S(IV) concentrations alter the rates of catalysis via ${\text{Fe}}^{3+}$ and ${\text{Mn}}^{2+}$, along with their synergistic catalysis, but did not explore a pH or temperature dependency. However, at similar pH ranges, S(IV) concentrations, and soluble ${\text{Fe}}^{3+}$ and ${\text{Mn}}^{2+}$ concentrations, Martin and Good (1991) found that their rate expression agreed well with that by Ibusuki and Takeuchi (1987). When Shao et al. (2019) implemented the Ibusuki–Takeuchi TMI-catalyzed $O_{2}$ oxidation rate expression in ALW in GEOS-Chem, they found that this pathway accounted for 67%–69% of ${\text{SO}}_{4}^{2-}$ formed over China (Shao et al., 2019). The presence of higher S(IV) concentrations, however, may warrant the use of a ${\text{SO}}_{4}^{2-}$ formation rate that takes into consideration faster rates of TMI catalysis (Martin and Good, 1991). The TMI-$O_{2}$ oxidation pathway from Martin and Good (1991) is used in the Base_Het simulation, and the TMI-$O_{2}$ oxidation pathway from Ibusuki and Takeuchi (1987) is used in the TMI_sens simulation to explore the range in ${\text{SO}}_{4}^{2-}$ formation possible by this pathway (Table 1). For both implementations of this pathway, ionic strength impacts were added as high ionic strength has been found to limit the TMI-catalyzed oxidation of S(IV) to ${\text{SO}}_{4}^{2-}$ (Martin and Hill, 1967, 1987).

While the TMI-catalyzed oxidation pathway can be a significant contributor to secondary ${\text{SO}}_{4}^{2-}$ formation, especially at low pH, some studies suggest ${\text{SO}}_{4}^{2-}$ formation during Beijing winter haze events may be dominated by the reaction of ${\text{SO}}_{2}$ and ${\text{NO}}_{2}$ in aerosol and/or cloud and fog water under mildly acidic or neutral conditions (Cheng et al., 2016; Wang et al., 2020; Yang et al., 2019). This oxidation pathway may increase in importance with increasing ionic strength (Cheng et al., 2016). Recent chamber work by Chen et al. (2019) found that increasing ionic strength increased the rate of secondary ${\text{SO}}_{4}^{2-}$ formation from ${\text{NO}}_{2}$ oxidation. To assess the potential impact ionic strength may have on this pathway, the ionic-strength-dependent ${\text{NO}}_{2}$ oxidation rate of Chen et al. (2019) was included in the $\text{TMI}\_{\text{NO}}_{2}$ sensitivity simulation (Table 1).

While the aforementioned pathways are pH dependent, ${\text{SO}}_{2}$ aqueous oxidation by $H_{2}O_{2}$ is pH independent for pH > 2 due to the opposing dependencies of the reaction rate coefficient and S(IV) solubility on pH (Clifton et al., 1988; Seinfeld and Pandis, 2016). This pathway has been studied extensively in dilute conditions representative of cloud droplets (Hoffmann and Calvert, 1985; Maaß et al., 1999; McArdle and Hoffmann, 1983). Maaß et al. (1999) found that oxidation of S(IV) by $H_{2}O_{2}$ increases with increased ionic strength and formulated a semi-empirical relationship between ionic strength and the reaction rate coefficient for the $S(\text{IV})-H_{2}O_{2}$ oxidation pathway (the upper limit of ionic strength in this study is 5 M). Field measurements during Chinese haze events have reported ionic strengths of aerosols ranging from 14–43M (Cheng et al., 2016; Fountoukis and Nenes, 2007). Liu et al. (2020) recently studied this pathway using aerosol flow reactors to determine an ionic-strengthbased enhancement factor that encapsulates the combined ionic strength effects on Henry’s law coefficients, dissociation, and condensed-phase kinetics. This study found that increasing ionic strength from 0 to 14M resulted in an order of magnitude increase in ${\text{SO}}_{4}^{2-}$ production rate (Liu et al., 2020).

$O_{3}$ is another important aqueous-phase oxidant of ${\text{SO}}_{2}$ with a reaction rate that increases with increasing pH (Maahs, 1983) and ionic strength (Lagrange et al., 1994). The ionic strength enhancement factor for this rate has been implemented with other ionic strength enhancement or inhibition factors in the All_Ionic sensitivity simulation (Table 1).

For the fine-mode aerosol, total gas and particle concentrations of key inorganic species are passed to the thermodynamic equilibrium model ISORROPIA II to calculate aerosol pH and ALW (Fountoukis and Nenes, 2007). In addition to the ALW associated with inorganic ions, ALW associated with organic aerosols is also estimated in CMAQ via hygroscopicity parameters (Pye et al., 2017).

### 2.3 Model base case and sensitivity simulations

Several CMAQ configurations were used here to understand the impacts of using heterogeneous sulfur chemistry, ionic strength, and alternative pseudo-first-order rate expressions. A base case CMAQ simulation (“base”) was completed using in-cloud ${\text{SO}}_{4}^{2-}$ formation from aqueous oxidation by $H_{2}O_{2}$, $O_{3}$, PAA, and MHP and via TMI-catalyzed $O_{2}$ of ${\text{SO}}_{2}$ and gas-phase oxidation of ${\text{SO}}_{2}$ by OH (Fahey et al., 2017; Sarwar et al., 2013).

To account for the impacts of heterogeneous sulfur chemistry in ALW, the Base_Het model simulation was completed for all domains (see “Model configuration”) using the aforementioned heterogenous reactive uptake parameterizations (Eqs. 1–2). Parameters from CMAQ’s KMT2 cloud chemistry model (Fahey and Roselle, 2019; Fahey et al., 2017) were used to calculate $k_{\text{chem}}$ (Table 1). An ionic strength inhibition term was added to the TMI-catalyzed $O_{2}$ pathway in aerosol water to account for the limiting effect of ionic strength on this pathway. The ionic strength was capped at $I_{\max}=2\;M$ to reflect experimental constraints (Martin and Good, 1991; Martin and Hill, 1987; Seinfeld and Pandis, 2016). For this pathway in both clouds and ALW, ${\text{Fe}}^{3+}$ was assumed to be 90% of dissolved Fe at night and 10% during daytime, with soluble fractions of Mn and Fe assumed to be 0.5 and 0.1 of total Fe and Mn respectively (Alexander et al., 2009). Other sources of less soluble Fe and Mn emissions, such as dust, are likely minimal given the snow cover for this domain and episode (Shao et al., 2019).

The $k_{\text{chem}}$ for the TMI-catalyzed $O_{2}$ pathway in the Base_Het case is neither temperature nor pH dependent (Martin and Good, 1991; Martin and Hill, 1987). Ibusuki and Takeuchi (1987) found both a pH and a temperature dependence on $k_{\text{chem}}$ for this pathway (Martin and Hill, 1987). To explore the effects of both a pH and a temperature dependence on the rate of a TMI-catalyzed sulfur oxidation pathway, a sensitivity simulation, TMI_sens, was run (Ibusuki and Takeuchi, 1987). Given that this $k_{\text{chem}}$ is reduced in colder temperatures, the TMI_sens run likely represents a lower bound on ${\text{SO}}_{4}^{2-}$ formation for this pathway during winter episodes. This $k_{\text{chem}}$ also uses the same solubility, dissociation, and ionic strength bounds as the TMI-catalyzed $O_{2}$ oxidation pathway used in the Base_Het simulation.

In the $\text{TMI}\_{\text{NO}}_{2}\_\text{sens}$ simulations, both the alternative $k_{\text{chem}}$ for the TMI-catalyzed $O_{2}$ pathway and an ionicstrength-dependent $k_{\text{chem}}$ for the ${\text{NO}}_{2}$ oxidation pathway in ALW are included (Chen et al., 2019; Ibusuki and Takeuchi, 1987). Both the TMI-$O_{2}$ and the ${\text{NO}}_{2}$ oxidation $k_{\text{chem}}$ values favor weakly acidic pH regimes (Cheng et al., 2016; Martin and Good, 1991). This sensitivity simulation was implemented to analyze potential competition between two pathways under their favorable pH conditions and conditions that are also characteristic of wintertime haze episodes (weakly acidic) (Cheng et al., 2016).

Ionic strength impacts on $k_{\text{chem}}$ for $H_{2}O_{2}$ and $O_{3}$ aqueous oxidation formation pathways were included on top of the previous modifications in the TMI_sens and $\text{TMI}\_{\text{NO}}_{2}\_\text{sens}$ model simulations (Chen et al., 2019; Ibusuki and Takeuchi, 1987; Maaß et al., 1999). Ionic strength adjustments were also included for S(IV) dissociation constants and Henry’s law coefficients for $H_{2}O_{2}$, $O_{3}$, and ${\text{SO}}_{2}$ in the All_Ionic simulations (Ali et al., 2014; Lagrange et al., 1994; Maaß et al., 1999; Millero et al., 1989; Seinfeld and Pandis, 2016) to analyze the combined effects of ionic strength on total modeled ${\text{PM}}_{2.5,\text{sulf}}$ aerosol.

In addition to the reactions shown in Table 1 (which are treated in both aerosol and cloud water), also included is S(IV) oxidation by peroxyacetic acid (PAA) and methyl hydroperoxide (MHP) in aerosol and cloud water and in-cloud S(IV) oxidation by ${\text{HNO}}_{4}$, OH, and ${\text{NO}}_{3}$ (Lee and Schwartz, 1983a; Lind et al., 1987; Martin, 1984). With the exception of the base case, which used CMAQ’s default AQCHEM cloud chemistry scheme, all other simulations used the KMT2 cloud chemistry scheme. KMT2 includes additional inorganic and organic chemistry compared to AQCHEM, including several additional S(IV) oxidation reactions as well as HMS formation and loss.

### 2.4 Model configuration

Results of the base and sensitivity simulations were compared for three different spatial domains: Fairbanks, Alaska; the Contiguous US (CONUS); and the Northern Hemisphere. Model simulations over Fairbanks and North Pole, Alaska, span two wintertime PM episodes (Episode 1 (E1): 25 January–11 February 2008 and Episode 2 (E2): 4–17 November 2008) with 2 d of spin-up and a horizontal resolution of 1.33 km. Model simulations over the northern hemispheric domain were performed for the winter season from December 2015–February 2016 with 2 months of spin-up and a horizontal resolution of 108 km following a standard US EPA configuration described by Mathur et al. (2017) and Appel et al. (2021). Model simulations over the CONUS domain were run for the months of January and July 2016 with 10 d of spin up and at a horizontal resolution of 12 km.

The Weather Research and Forecasting (WRF) model (Skamarock et al., 2008) was used to develop meteorology on all three domains. The Fairbanks WRF case follows the original configuration and case study of Gaudet et al. (2010, 2012) for Fairbanks, AK. This older WRF simulation was updated from WRFv3.3 (Gaudet et al., 2010, 2012) to WRFv4.1.1. with all geophysical and meteorological inputs reprocessed for compatibility with the more recent version of WRF and using the Mellor–Yamada–Nakanishi–Niino 2.5-order (MYNN2.5) closure scheme (Nakanishi and Niino, 2009). Sensitivity testing found performance improvements when updating the planetary boundary layer model change from the Mellor–Yamada–Janjić (MYJ) scheme to MYNN2.5. Evaluations also showed that the WRFv4.1.1 configuration captured the extreme temperature variations in these cases well, with 2 m temperature root mean square error (RMSE) of the order of 2–3 K, which is within historical benchmarks for complex geographical areas that are more difficult to model (Kemball-Cook et al., 2005). For the CONUS, meteorological inputs were sourced from WRFv4.1.1, and CMAQ inputs were processed with the Meteorology-Chemistry Interface Processor (MCIP; Appel et al., 2017; Otte and Pleim, 2010) version 5.0.

The emissions inputs for the two Fairbanks wintertime PM episodes were based on inputs provided by the Alaska Department of Environmental Conservation (ADEC, 2014). These emission estimates were from the base year (2008) used for the Fairbanks PM_2.5_ moderate state implementation plan (SIP) and represent the best available emission estimates for the two time periods. Table 5.6-3 from Sect. 5.06 of ADEC (2014) provides a summary of the methods and inputs used to develop this emission inventory. For this model setup, we used the same inventory inputs and scripts and only updated the speciation for CMAQv5.3.2. The emission inventories for the northern hemispheric domain follow the same setup as described in Appel et al. (2021), where anthropogenic emissions were sourced from the 2010 Hemispheric Transport of Air Pollution version 2 (Janssens-Maenhout et al., 2015), biogenic emissions were sourced from the Model of Emissions of Gases and Aerosols from Nature (Guenther et al., 2012), soil and lightning NO were sourced from the Global Emissions Initiative (http://www.geiacenter.org, last access: 3 May 2021), biomass burning emissions were sourced from the Fire Inventory from the National Center for Atmospheric Research (Wiedinmyer et al., 2011), and on-road and non-road emissions were developed using the Motor Vehicle Emission Simulator v2014a. The emission inventories for the CONUS domain were sourced from the 2016v7.2 (beta and Regional Haze) modeling platform (Appel et al., 2021).

Gas-phase chemistry was simulated using the CB6r3 mechanism (Luecken et al., 2019), and aerosol dynamics were simulated using the aero7 module. Boundary conditions for the Fairbanks domain were sourced from EPA’s Air QUAlity TimE Series (EQUATES) project for 2008 (USEPA, 2021). The sulfur-tracking method (STM) (Fahey and Roselle, 2019) was extended to include the new heterogeneous sulfur chemical pathways in order to track the contributions of each chemical reaction, primary emissions, and initial and boundary conditions to modeled ${\text{SO}}_{4}^{2-}$ (Appel et al., 2021).

### 2.5 Sulfate and ${\text{PM}}_{2.5,\text{sulf}}$ observations

The model predictions were evaluated against available observations. While most monitoring networks report measurements for PM_2.5_ or ${\text{PM}}_{1}\;{\text{SO}}_{4}^{2-}$, recent studies have indicated hydroxymethanesulfonate may be included in those observations (Dovrou et al., 2019; Ma et al., 2020; Moch et al., 2018; Song et al., 2019). Based on these findings, we compare measured ${\text{SO}}_{4}^{2-}$ to modeled ${\text{PM}}_{2.5,\text{sulf}}$:

(4) $${\text{PM}}_{2.5,\text{sulf}}(\frac{\mu g}{m^{3}})={\text{SO}}_{4}^{2-}+\text{HMS}\times \frac{{\text{MW}}_{{\text{SO}}_{4}^{2-}}}{{\text{MW}}_{\text{HMS}}}.$$

${\text{PM}}_{2.5}{\text{SO}}_{4}^{2-}$ observations in Fairbanks during 2008 were obtained from ADEC’s Thermo Electron Partisol 2000 single-channel instrument w/SCC monitor 020900010 (State Office Building in Fairbanks Alaska; 64.840672, −147.722461) (USEPA, 2024a). ${\text{SO}}_{4}^{2-}$ observations for the 2016 hemispheric domain were sourced from the United States Environmental Protection Agency (US EPA) Air Quality System (AQS) monitoring network; the Canadian National Air Pollution Surveillance (NAPS) monitoring network; the European Monitoring and Evaluation Programme (EMEP) monitoring network; and one monitor at Tsinghua University in Beijing, China (ECCC, 2022; Ma et al., 2020; Zhang et al., 2021a; Tørseth et al., 2012; USEPA, 2022). Modeled ${\text{PM}}_{2.5,\text{sulf}}$ concentrations and measured ${\text{SO}}_{4}^{2-}$ from the AQS and NAPS networks were cast in units of micrograms sulfur per meter cubed (μg S m^−3^) to match the measurement units used in the EMEP and Tsinghua University ${\text{SO}}_{4}^{2-}$ measurements.

## 3 Results

### 3.1 Modeled particulate sulfur enhancement during dark and cold PM episodes in Fairbanks and North Pole, AK

#### 3.1.1 Aerosol sulfur enhancements during a wintertime haze event (Episode 1 (E1))

**Figure 1.**
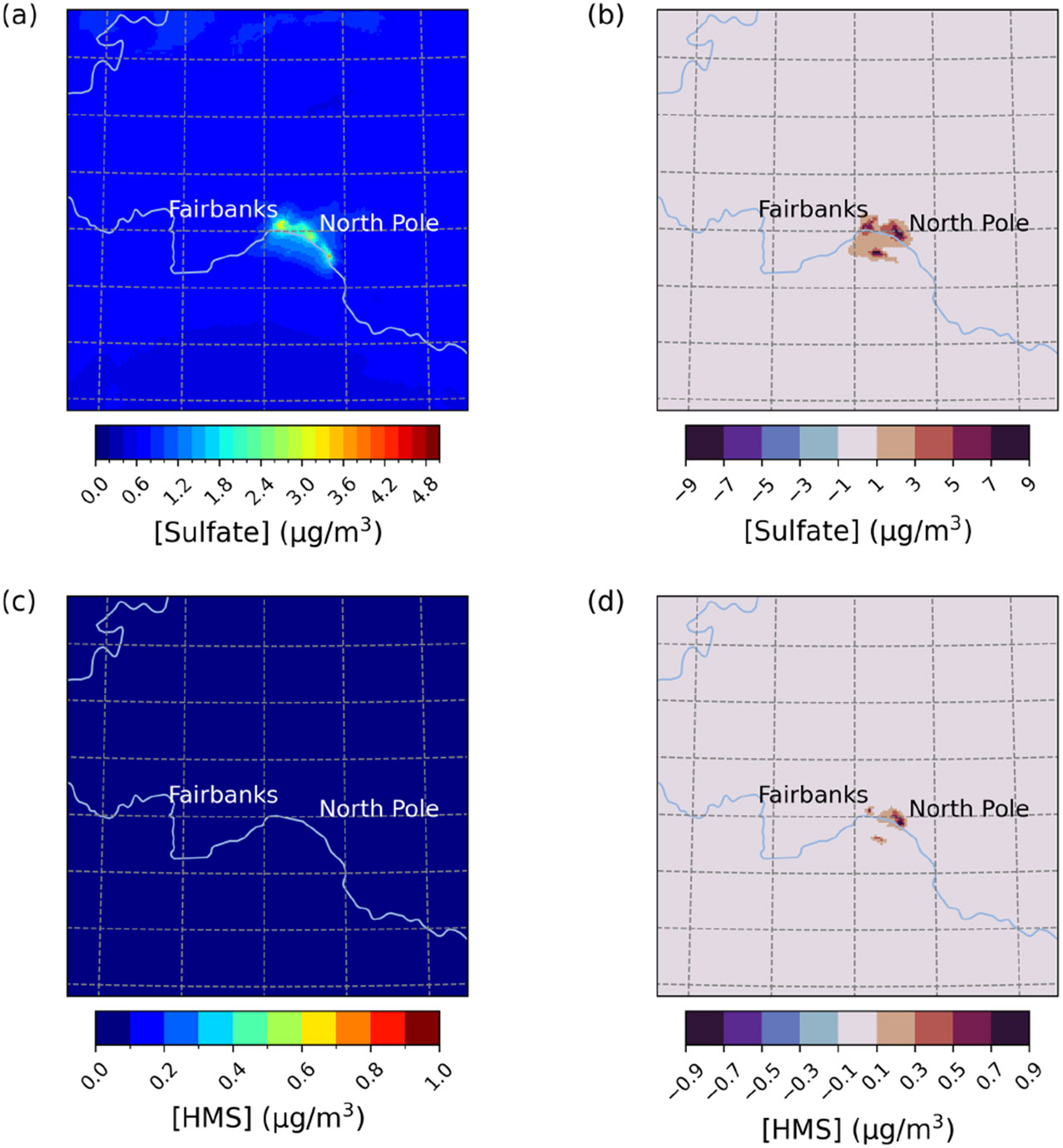
Episode average sulfate **(a)** and HMS **(c)** concentrations in the base simulation along with daily max differences in sulfate **(b)** and HMS **(d)** concentrations between the Base_Het and base CMAQ simulations over Fairbanks and North Pole, AK, for Episode 1 (from 25 January to 11 February 2008). HMS formation was not included in base CMAQ (i.e., HMS = 0 in the base simulation). The domain size is 264.67 km by 264.67 km with a grid cell resolution of 1.33 km by 1.33 km.

The base simulation average E1 sulfate concentrations around Fairbanks and North Pole, AK, are ~2–3.5 mg m^−3^ (Fig. 1a and c). Compared to the base, the Base_Het simulation leads to increased ${\text{PM}}_{2.5,\text{sulf}}$ predictions concentrated around the cities of Fairbanks and North Pole as well as the region south of the Tanana River (Fig. 1b, d). The additional heterogeneous chemistry in the Base_Het simulation contributes up to an additional 11 μg m^−3^ of maximum daily ${\text{PM}}_{2.5,\text{sulf}}$ compared to the base simulation in the region south of the Tanana River. Maximum daily differences are defined as

(5) $$\text{Maximum daily differences}=\;\;\max({\text{Avg}}_{\text{daily},2}-{\text{Avg}}_{\text{daily},1}).$$

Enhancements in ${\text{PM}}_{2.5,\text{sulf}}$ concentrations for the Base_Het simulation are mainly driven by increases in ${\text{SO}}_{4}^{2-}$ concentrations (increasing up to 10.9 μg m^−3^ for daily maximum differences across the entire domain), with smaller impacts from HMS (increasing up to ~1 μg m^−3^ for daily maximum differences across the entire domain). HMS concentrations are enhanced more in North Pole than Fairbanks, coinciding with higher HCHO emissions (Fig. S1 in the Supplement) from residential wood combustion combined with high co-located ${\text{SO}}_{2}$ emissions (from home-heating oil) along with lower temperatures (ADEC, 2017).

**Figure 2.**
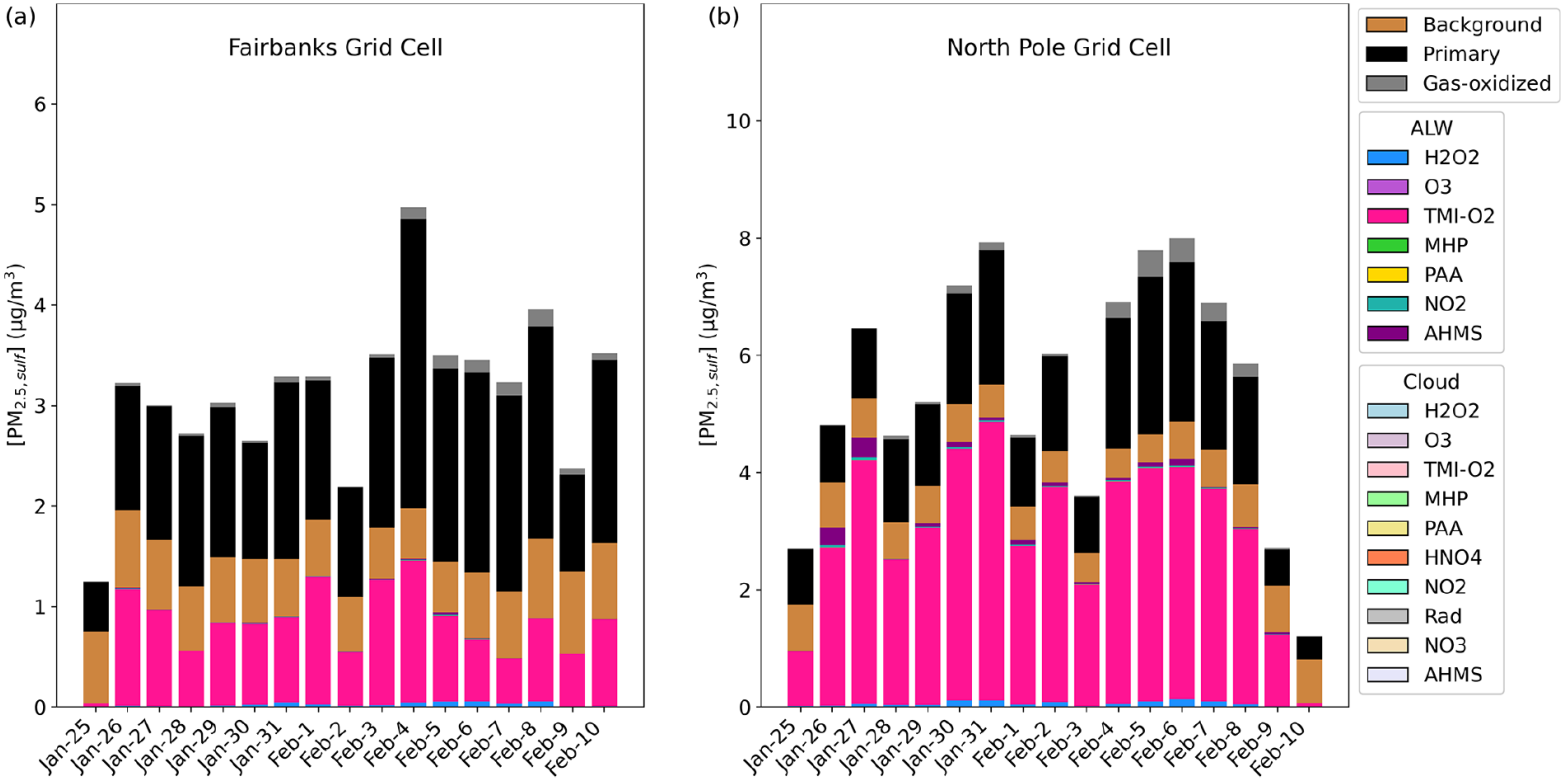
Particulate sulfur process/chemistry contributions and speciation (${\text{SO}}_{4}^{2-}$ and HMS) in downtown Fairbanks **(a)** located at the State Office Building (64.84° N, 147.72° W; grid cell 108, 93) and North Pole **(b)** located at 64.76° N, 147.34° W (grid cell 122, 86) for Episode 1 (E1) speciated by source and/or formation pathway. Secondary aqueous formation of ${\text{PM}}_{2.5,\text{sulf}}$ is divided into two categories: ALW and cloud, where ALW pathways represent the heterogeneous sulfur chemistry added in this study.

Out of all of the secondary ${\text{PM}}_{2.5,\text{sulf}}$ formation pathways that are enhanced during dark, cold conditions (TMI-catalyzed $O_{2}$, ${\text{NO}}_{2}$, and the formation of HMS), the leading secondary ${\text{SO}}_{4}^{2-}$ formation pathway in Base_Het is the TMI-catalyzed $O_{2}$ oxidation pathway in ALW (Fig. 2). The first-order condensed-phase rate constant ($k_{\text{chem}}$) of this pathway is lower than that of $k_{\text{chem}}$ for ${\text{NO}}_{2}$ by almost 2 orders of magnitude for average modeled conditions characteristic of Fairbanks and North Pole for E1 (aerosol pH = 3.83, [Fe(III)] = 0.24 M, [Mn (II)] = 0.002 M, $[{\text{SO}}_{2}]=20\;\text{ppb}$, $[{\text{NO}}_{2}]=20\;\text{ppb}$, $[{\text{SO}}_{4}^{2-}]=3\;\mu g\;m^{-3}$, $[\text{ALW}]=6\;\mu g\;m^{-3}$, and Temp = 243 K) (Fig. S2) and is ~1 order of magnitude higher than that for HMS formation in ALW. Despite the ${\text{NO}}_{2}$ $k_{\text{chem}}$ being higher, the TMI-catalyzed $O_{2}$ heterogeneous rate of sulfate formation is dependent upon ${\text{SO}}_{2}$ partitioning into the particle, as Fe and Mn are both aerosol species, and simulated dark conditions reduce the conversion of ${\text{Fe}}^{3+}$ to ${\text{Fe}}^{2+}$ from daytime photochemical reactions (Alexander et al., 2009; Rao and Collett, 1998; Shao et al., 2019). The effective Henry’s law coefficient for ${\text{SO}}_{2}$ increases with pH, while Henry’s law coefficient for ${\text{NO}}_{2}$ remains low across the pH spectrum. This and a higher mass accommodation coefficient (by ~2 orders of magnitude) for ${\text{SO}}_{2}$ compared to ${\text{NO}}_{2}$ contribute to the TMI-catalyzed $O_{2}$ pathway outcompeting the ${\text{NO}}_{2}$ pathway for this model configuration. The TMI-catalyzed $O_{2}$ heterogeneous reactive uptake pathway also outcompetes the $H_{2}O_{2}$ and $O_{3}$ heterogeneous reactive uptake pathways due to low photochemical activity with the dark conditions of this domain and episode.

The formation of HMS is higher in North Pole, which can be colder than Fairbanks by up to ~5 °C. Higher modeled HCHO emissions in North Pole (Fig. S1) along with colder temperatures increase the partitioning of HCHO into existing particles. This effect and the increase in HMS formation are more pronounced in the TMI_sens simulation (Fig. S3a and b).

Compared to $k_{\text{chem}}$ used in Base_Het, the alternative $k_{\text{chem}}$ in TMI_sens for the TMI-catalyzed $O_{2}$ pathway is ~2 orders of magnitude lower under average conditions characteristic of this episode (Fig. S2), with the extremely cold temperatures decreasing the TMI-catalyzed $O_{2}$ oxidation $k_{\text{chem}}$. This ultimately results in a slower conversion of S(IV) species to ${\text{SO}}_{4}^{2-}$ and subsequent competition with HMS formation, which increases with colder temperatures (Fig. S4). Maximum daily average enhancements in modeled ${\text{PM}}_{2.5,\text{sulf}}$ concentrations in the TMI_sens simulation reach up to 25 μg m^−3^ at a grid cell in North Pole and are mostly attributed to high HMS concentrations (Fig. S3a and b). Formation rates of HMS are also dependent on aerosol pH, which can be higher in North Pole than in Fairbanks (Fig. S4). Higher pH increases the conversion of ${\text{HSO}}_{3}^{-}$ to ${\text{SO}}_{3}^{2-}$, and the rate constant for HCHO reaction with ${\text{SO}}_{3}^{2-}$ is 5 orders of magnitude higher than that of the reaction with ${\text{HSO}}_{3}^{-}$ (Boyce and Hoffmann, 1984). Lower ${\text{SO}}_{4}^{2-}$ production rates in TMI_sens and lower modeled aerosol acidity compared to Base_Het likely contribute to the higher HMS formation (and loss) rates seen in the TMI_sens simulation. It is important to note that while aerosol acidity is modified by aqueous-phase formation of ${\text{SO}}_{4}^{2-}$, it is not modified by the formation of HMS in CMAQ.

When implementing an ionic-strength-dependent ${\text{NO}}_{2}$ rate expression (Chen et al., 2019) on top of this alternative TMI-catalyzed $O_{2}$ rate expression in the $\text{TMI}\_{\text{NO}}_{2}\_\text{sens}$ model simulation, the ${\text{NO}}_{2}$ oxidation pathway outcompeted the formation of HMS (Fig. S3). While this model simulation compared well with the Base_Het model simulation in daily averaged ${\text{PM}}_{2.5,\text{sulf}}$ concentrations, it predicted significantly lower ${\text{PM}}_{2.5,\text{sulf}}$ concentrations than TMI_sens in North Pole due to a reduction in HMS predicted. Compared to downtown Fairbanks, North Pole has lower ${\text{NO}}_{2}$ emissions, but the switch to the ionic-strength-dependent reaction rate still leads to higher ${\text{SO}}_{4}^{2-}$ production in North Pole compared to TMI_sens.

The ALL_Ionic model simulation resulted in similar ${\text{PM}}_{2.5,\text{sulf}}$ predictions and pathway contributions to the $\text{TMI}\_{\text{NO}}_{2}\_\text{sens}$ simulation for this episode (Fig. S3). In both Fairbanks and North Pole, ${\text{SO}}_{4}^{2-}$ formation attributed to heterogeneous reactive uptake via the $H_{2}O_{2}$ oxidation pathway increased slightly. For ionic strength factor calculations, ionic strength is capped at the maximum ionic strength considered in the experiments the parameterizations are based on (~5–6 M) (Ali et al., 2014; Millero et al., 1989). The modeled ionic strength of ALW is typically at or above the maximum experimental ionic strength when deriving the effective Henry’s law coefficients, dissociation coefficients, and $k_{\text{chem}}$ for the $H_{2}O_{2}$ oxidation pathway – leading to expected higher dissolution and kinetics for this pathway. HMS concentrations for this model simulation also increased slightly in comparison to $\text{TMI}\_{\text{NO}}_{2}\_\text{sens}$. Assuming excess of dark oxidant precursors (TMI, ${\text{NO}}_{2}$, and HCHO) and maximum ionic strength, the leading higher HMS production rate in ALL_Ionic is likely due to a slightly higher range of aerosol pH (~2–5.5) compared to the Base_Het and $\text{TMI}\_{\text{NO}}_{2}\_\text{sens}$ simulations (Fig. S3d).

#### 3.1.2 Aerosol sulfur enhancements with liquid cloud events (Episode 2 (E2))

Sulfate and HMS are known to form efficiently in cloud and fog droplets (Altwicker and Nass, 1983; Boyce and Hoffmann, 1984; Calvert et al., 1978; Clifton et al., 1988; Ibusuki and Takeuchi, 1987; Lee and Schwartz, 1983a; Martin and Good, 1991; McArdle and Hoffmann, 1983). In E1, there was minimal cloud or fog liquid water simulated; however, during E2 (4–11 November 2008), there were some periods where cloud/fog chemistry impacts on ${\text{PM}}_{25,\text{sulf}}$ formation were evident.

**Figure 3.**
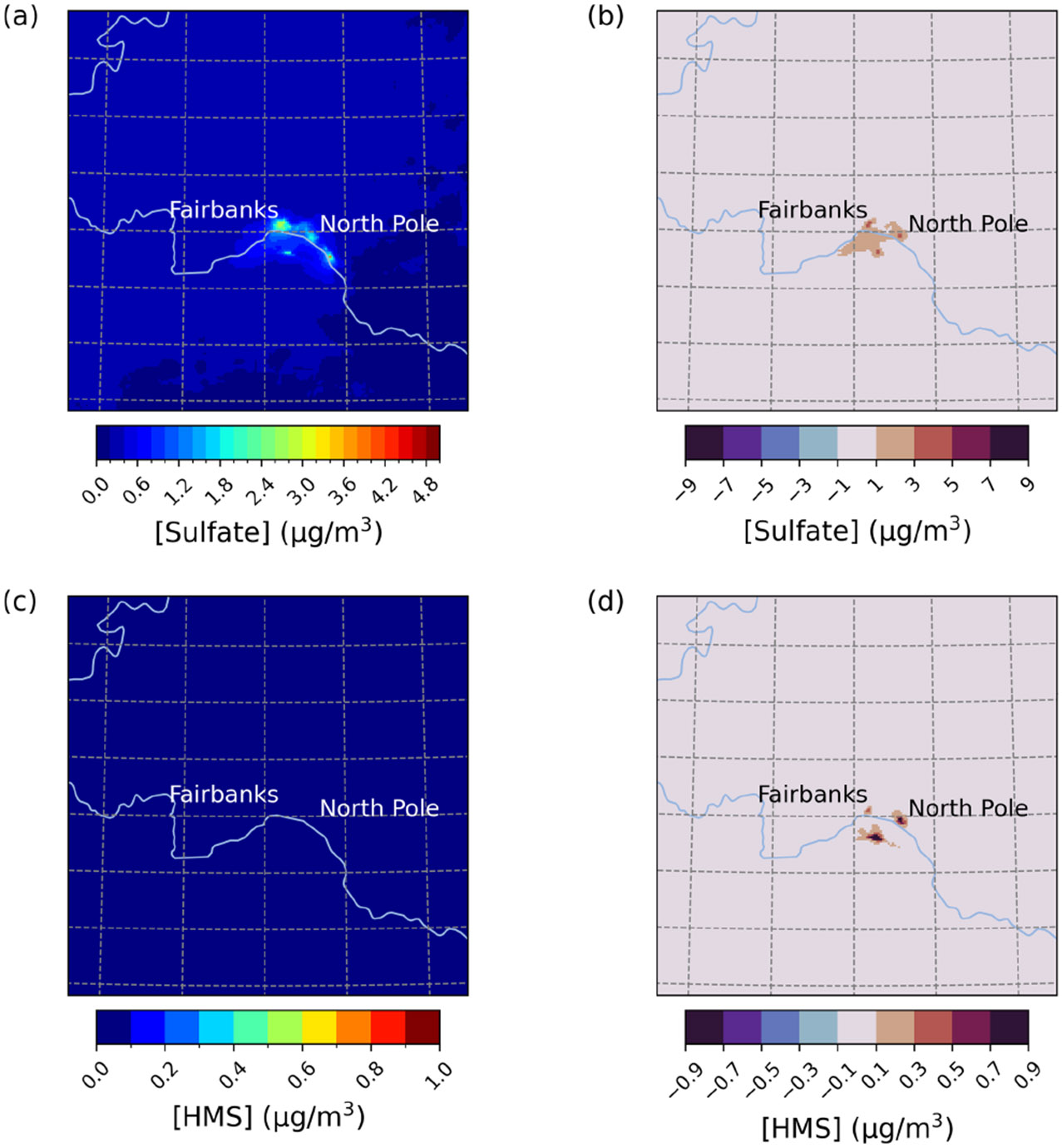
Episode average sulfate **(a)** and HMS **(c)** concentrations in the base simulation along with daily max differences in sulfate **(b)** and HMS **(d)** concentrations between the Base_Het and base CMAQ simulations over Fairbanks and North Pole, AK, for Episode 2 (from 4 to 17 November 2008). HMS formation was not included in base CMAQ (i.e., HMS = 0 in the base simulation). The domain size is 264.67 km by 264.67 km with a grid cell resolution of 1.33 km by 1.33 km.

Compared to E1, ${\text{PM}}_{2.5,\text{sulf}}$ concentration enhancements were lower overall during E2. Differences between Base_Het and base simulations, however, are appreciable during this episode, with ${\text{PM}}_{2.5,\text{sulf}}$ increasing up to 4.6 μg m^−3^ across the entire domain (daily maximum difference) (Fig. 3). Enhancements in ${\text{PM}}_{2.5,\text{sulf}}$ are mainly driven by increased ${\text{SO}}_{4}^{2-}$ formation in and around Fairbanks and North Pole; however, simulated HMS concentrations reached up to 4.4 μg m^−3^ south of the Tanana River (daily maximum) for this episode. Note that the base simulation included some contributions from in-cloud S(IV) oxidation (i.e., five S(IV) oxidation reactions from CMAQ’s default cloud chemistry mechanism (AQCHEM)), while Base_Het included the additional in-cloud chemical reactions from the KMT2 cloud chemistry module.

**Figure 4.**
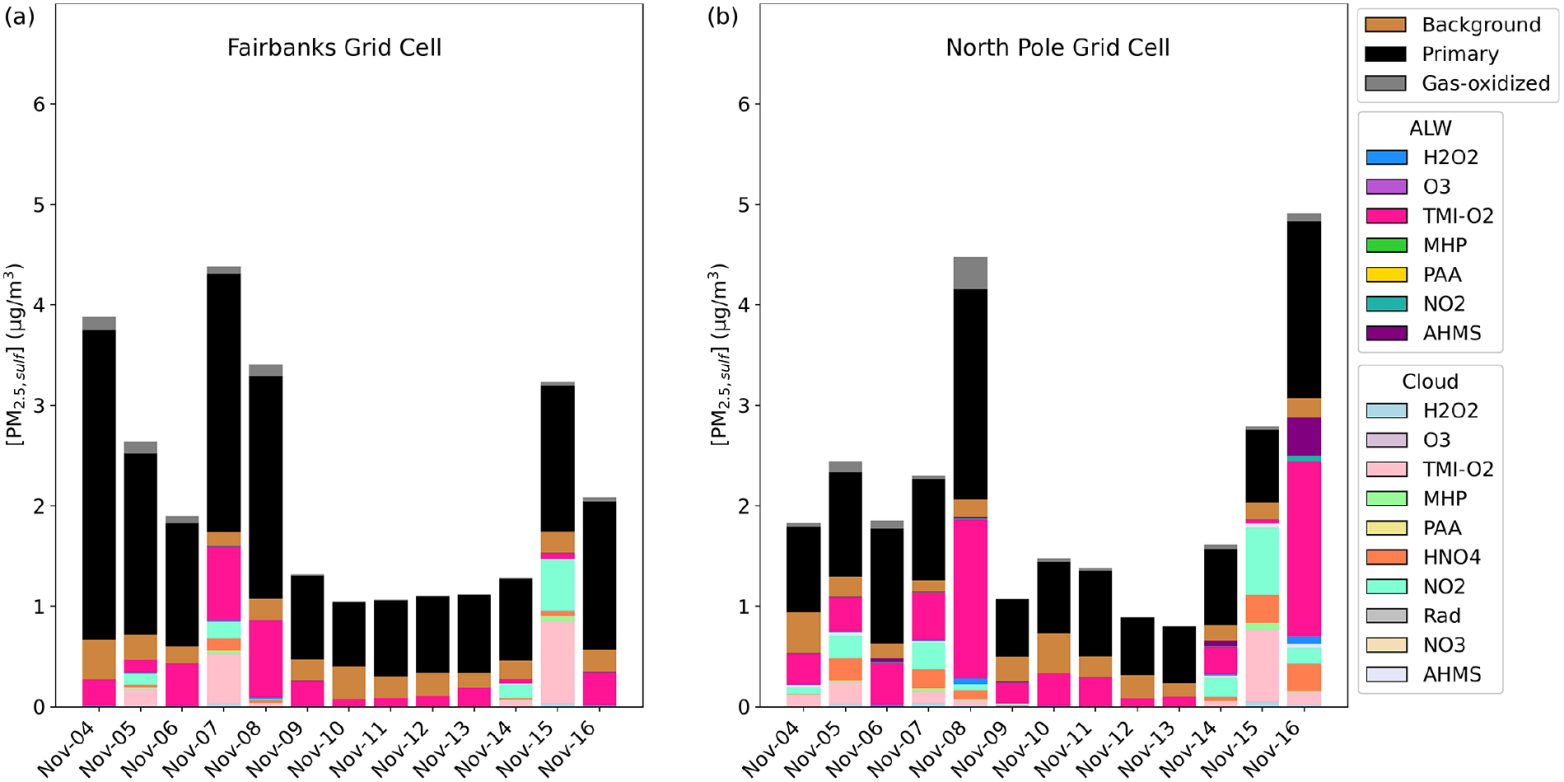
Particulate sulfur process/chemistry contributions and speciation (${\text{SO}}_{4}^{2-}$ and HMS) in downtown Fairbanks **(a)** located at the State Office Building (64.84° N, 147.72° W; grid cell 108, 93) and North Pole **(b)** located at 64.76° N, 147.34° W (grid cell 122, 86) for Episode 2 speciated by source and/or formation pathway. Secondary aqueous formation of ${\text{PM}}_{2.5,\text{sulf}}$ is divided into two categories: ALW and cloud, where ALW pathways represent the heterogeneous sulfur chemistry added in this study.

In the Base_Het simulation of Episode 2, ${\text{PM}}_{2.5,\text{sulf}}$ was formed in both ALW and cloud liquid water (Fig. 4). Similar to Episode 1, the leading secondary formation pathway in downtown Fairbanks was the TMI-catalyzed $O_{2}$ pathway in both ALW and cloud water. This formation pathway split between the ALW and cloud formation pathways when modeled fog water content was around 0.025–0.05 g m^−3^ for 5 and 7 November; however, it was completely overtaken by the cloud formation pathway on 15 November when fog water content was > 0.175 g m^−3^ (at both Fairbanks and North Pole), highlighting that when surface clouds or fog water is present, reactions in cloud water can compete with those in aerosol water. In North Pole, the leading ALW ${\text{PM}}_{2.5,\text{sulf}}$ formation pathway was also the TMI-catalyzed $O_{2}$ oxidation pathway, with contributions from this pathway in cloud water as well. Sulfate formed via S(IV) oxidation by ${\text{NO}}_{2}$ in cloud water was slightly higher in North Pole than downtown Fairbanks for all cloud events, even though ${\text{NO}}_{2}$ emissions were higher in downtown Fairbanks (Fig. S5). This could be due to higher cloud/fog pH in North Pole compared to downtown Fairbanks, as this pathway is known to favor pH > 5 (Clifton et al., 1988; Lee and Schwartz, 1983a; Littlejohn et al., 1993; Sarwar et al., 2013). HMS formation in ALW is also present in North Pole on the last day of Episode 2, corresponding with temperatures around −28 °C and aerosol pH ~5.

HMS contribution to ${\text{PM}}_{2.5,\text{sulf}}$ concentrations is further increased on 16 November in North Pole in the TMI_sens model simulation (Fig. S7b). Despite this increase in HMS, total ${\text{PM}}_{2.5,\text{sulf}}$ predicted in this simulation is lower than in the Base_Het simulation for most of the episode. The compensation of the HMS formation pathway for the TMI-catalyzed $O_{2}$ formation pathway is not as significant for this episode despite similar HCHO emissions (Fig. S5) and is likely due to higher temperatures (~+15 °C warmer than E1).

#### 3.1.3 Improved model performance for ${\text{PM}}_{2.5,\text{sulf}}$ in Fairbanks

**Figure 5.**
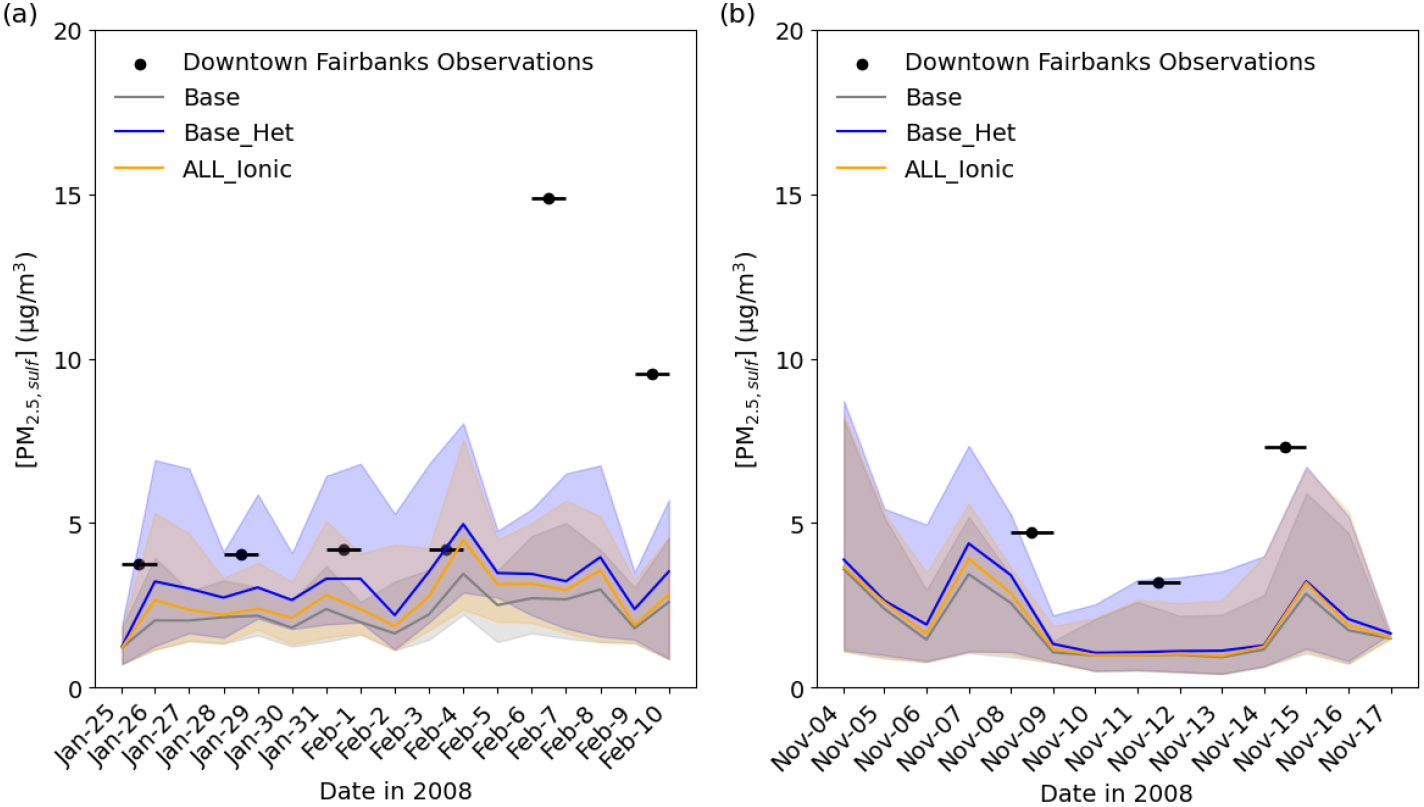
Time series comparing modeled ${\text{PM}}_{2.5,\text{sulf}}$ concentrations to measured ${\text{SO}}_{4}^{2-}$ concentrations at the State Office Building in downtown Fairbanks (64.84° N, 147.72° W; grid cell 108, 93) for Episode 1 **(a)** and Episode 2 **(b)**. The lines represent daily average modeled values, and the shading represents hourly maximum and minimum concentrations for each day. The black lines represent the sampling period of the monitor measurements, with black circles representing the mid-point of the 24 h sampling period.

Daily average ${\text{PM}}_{2.5,\text{sulf}}$ concentrations for the base and Base_Het simulations were compared with 24h ${\text{SO}}_{4}^{2-}$ measurements taken every third day at the State Office Building in downtown Fairbanks (Fig. 5) (USEPA, 2024a).

With the inclusion of heterogeneous sulfur chemistry in CMAQ (Base_Het), the mean bias in ${\text{PM}}_{2.5,\text{sulf}}$ improved by ~0.62 μg m^−3^ during E1 and by ~0.36 μg m^−3^ during E2, reducing the model bias by up to ~1 μg m^−3^ during E1 and up to ~0.85 μg m^−3^ during E2. CMAQ still underpredicts ${\text{PM}}_{2.5,\text{sulf}}$ observations for both episodes by ~11 and 7 μg m^−3^, particularly during E1 for 6 and 9 February respectively. PM_2.5_ speciation measurements during both episodes were only available for a single monitor located in downtown Fairbanks, and therefore we were not able to assess model performance in North Pole, where modeled ${\text{PM}}_{2.5,\text{sulf}}$ concentrations can be higher by up to ~5–27 μg m^−3^ (daily maximum difference) for E1 and ~2–2.4 μg m^−3^ (daily maximum difference) for E2. Although there were no measurements to compare with modeled North Pole results, higher ${\text{PM}}_{2.5,\text{sulf}}$ enhancements in North Pole are consistent with higher PM_2.5_ concentrations in North Pole compared to Fairbanks (ADEC, 2017).

### 3.2 Modeled ${\text{PM}}_{2.5,\text{sulf}}$ formation enhancements over the Northern Hemisphere in winter

To investigate model performance with the addition of heterogeneous sulfur chemistry for other locations and time periods that experience dark and cold conditions, Hemispheric CMAQ (HCMAQ) was run over the Northern Hemisphere from October 2015 to February 2016 (with the first 2 months as model spin-up) for the same base and sensitivity simulations as above. ${\text{PM}}_{25,\text{sulf}}$ in China was of interest in this domain, given its haze events. High secondary and heterogeneously formed ${\text{SO}}_{4}^{2-}$ and HMS have been documented to coincide with the high ${\text{SO}}_{2}$ emissions, high PM loadings, and high ALW during Chinese haze events (Cheng et al., 2016; Elser et al., 2016; Li et al., 2017; Ma et al., 2020; Peng et al., 2021; Wang et al., 2016). Winter episode average modeled ${\text{SO}}_{2}$ over northeastern China and other parts of China can be > 20 ppb, episode average modeled PM_2.5_ can be > 80 μg m^−3^, and episode average ALW can be > 80 μg m^−3^ in this region (Fig. S8).

**Figure 6.**
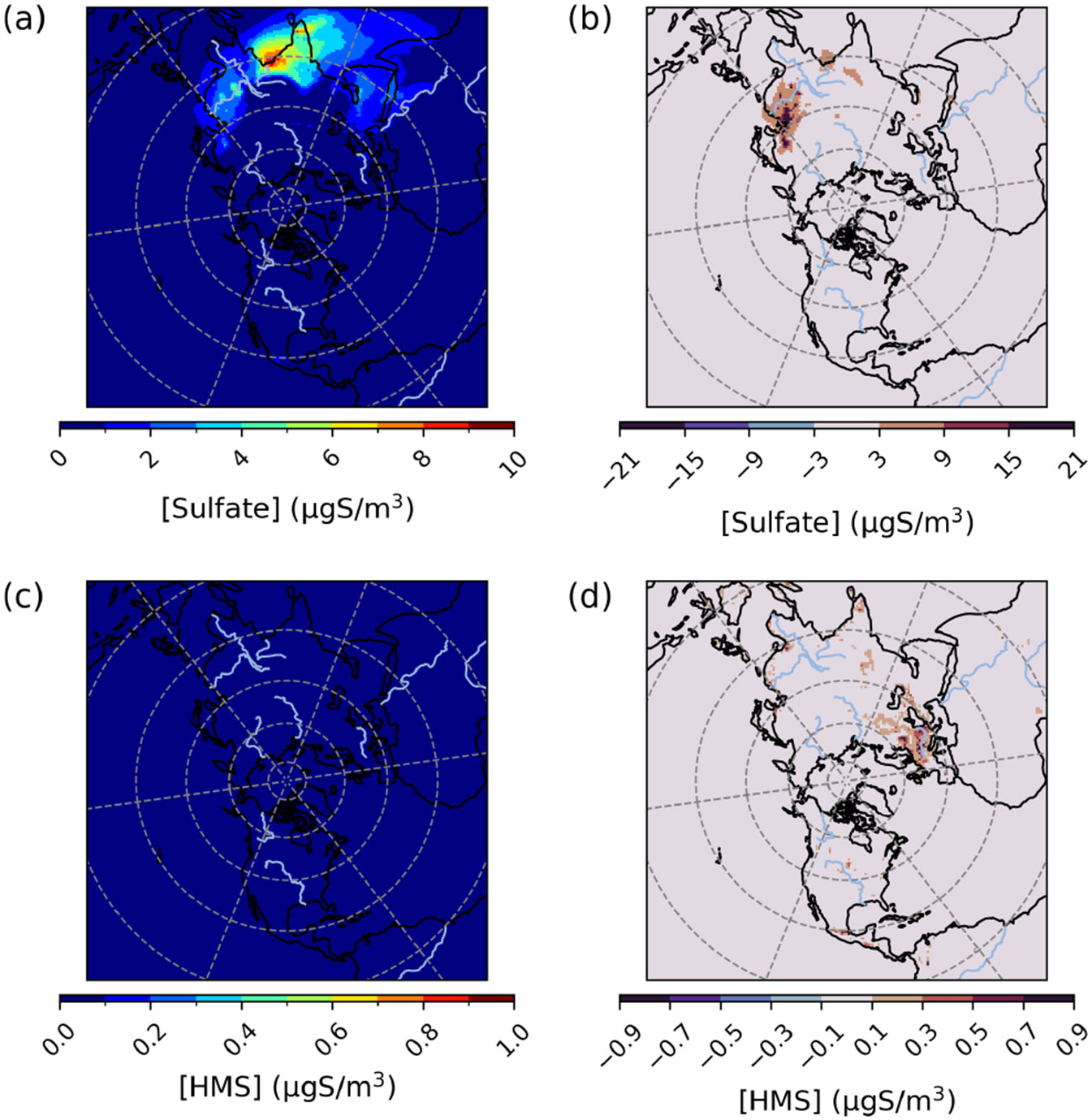
Episode average sulfate **(a)** and HMS **(c)** concentrations in the base simulation along with maximum daily differences in sulfate **(b)** and HMS **(d)** concentrations between the Base_Het and base CMAQ simulations over the Northern Hemisphere for a wintertime episode (from 1 December 2015 to 29 February 2016). HMS formation was not included in base CMAQ. Differences are cast in micrograms of sulfur per meter cubed to be consistent with measurement units from EMEP.

Maximum enhancements in ${\text{PM}}_{2.5,\text{sulf}}$ for the Base_ Het simulation occurred largely in the North China Plain and northeastern China, with some notable enhancement over India as well (Fig. 6), and led to a maximum daily increase in ${\text{PM}}_{2.5,\text{sulf}}$ by up to ~54μgS m^−3^ at a grid cell in southern China (27.1651° N, 107.5234° W; grid cell 149, 67) for this winter period (Fig. 6).

This maximum daily enhancement is due almost entirely to an increase in predicted ${\text{SO}}_{4}^{2-}$ (max daily concentration of 53 μgS m^−3^ at this same location). On average, ${\text{SO}}_{4}^{2-}$ can increase by up to ~9 μgS m^−3^ over a grid cell in northeastern China (45.6698° N, 127.9877° W; grid cell 122, 64). HMS contributions to ${\text{PM}}_{2.5,\text{sulf}}$ for the Base_Het run over this domain were less significant, with a maximum daily concentration of ~2.6 μgS m^−3^ in a grid cell near Tehran, Iran (36.7976° N, 51.6208° W; grid cell 137, 119).

For the TMI_sens simulation, maximum daily ${\text{PM}}_{2.5,\text{sulf}}$ concentrations increased from the base simulation by up to ~33 μgS m^−3^ and on average up to ~7 μgS m^−3^ at a grid cell in Hebei, China (39.8603° N, 119.2348° W; grid cell 131, 65) (a reduced episodic enhancement in comparison to the Base_Het simulation). This maximum enhancement is also almost entirely attributed to ${\text{SO}}_{4}^{2-}$ increases. However, HMS concentrations in the TMI_sens simulation contributed up to ~2.8 μgS m^−3^ (in maximum daily concentrations) in a grid cell in northeastern China (45.6698° N, 127.9877° W; grid cell 122, 64) (Fig. S9). Predicted ${\text{PM}}_{2.5,\text{sulf}}$ concentrations in the $\text{TMI}\_{\text{NO}}_{2}\_\text{sens}$ and All_Ionic simulations were similar to the TMI_sens simulation for both predicted ${\text{SO}}_{4}^{2-}$ and predicted HMS concentrations, with the exception of not reproducing as high HMS concentrations in northeastern China (Fig. S9).

**Figure 7.**
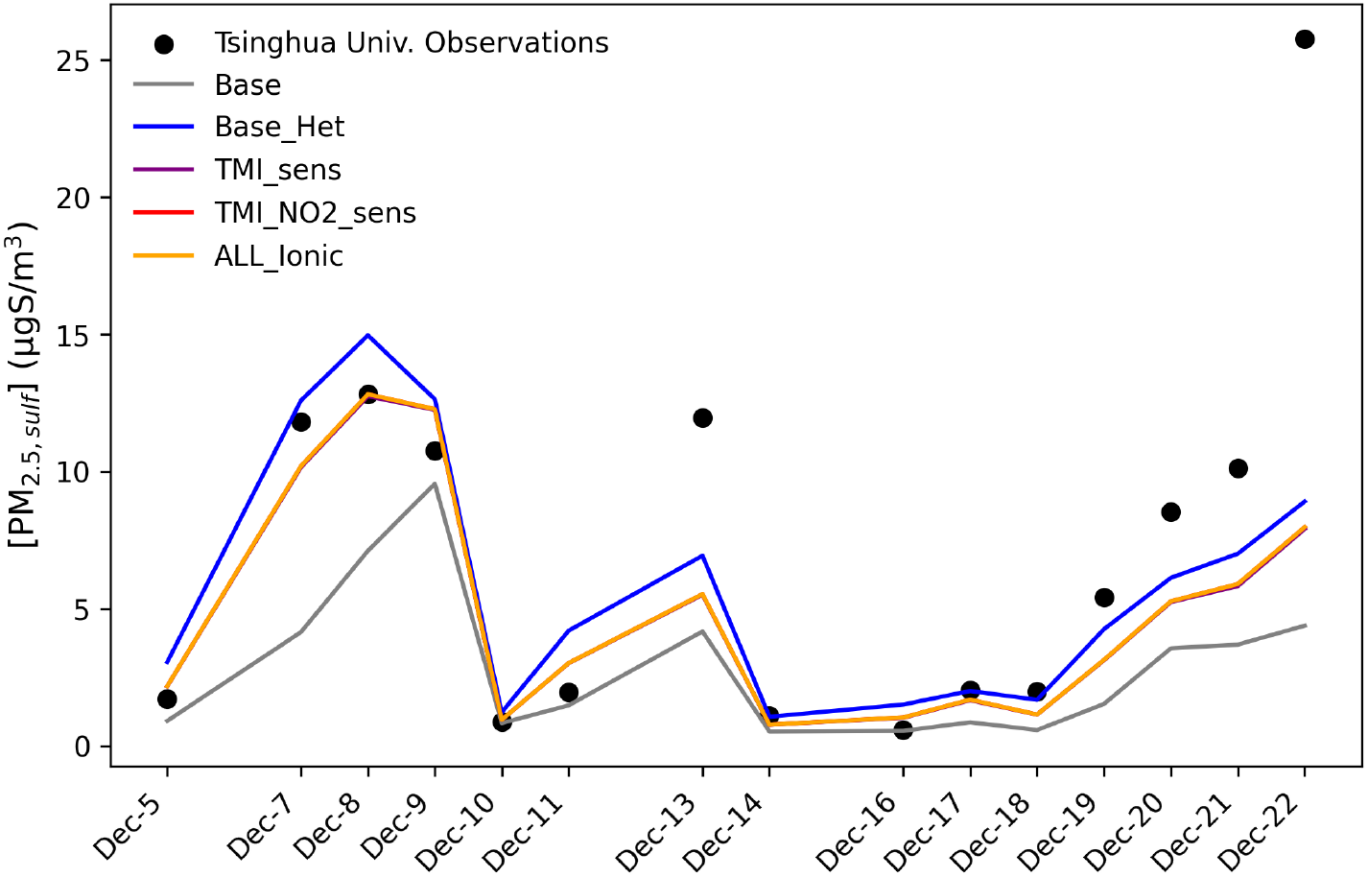
Model–measurement comparisons of ${\text{PM}}_{2.5,\text{sulf}}$ across all model runs for HCMAQ at a grid cell over Tsinghua University in Beijing, China, from 5 to 22 December 2015. Observations are sourced from Zhang et al. (2021b).

The addition of heterogenous sulfur chemistry decreased the model bias during an extreme haze event on the HCMAQ domain as well. Modeled ${\text{PM}}_{2.5,\text{sulf}}$ concentrations from a grid cell over Beijing were compared to sulfate measurements at Tsinghua University in Beijing (Zhang et al., 2021a) (Fig. 7).

The Base_Het and all additional sensitivity runs predicted higher ${\text{PM}}_{2.5,\text{sulf}}$ at this grid cell than the base model simulation and reduced modeled mean bias by 2.9 μgS m^−3^ (model mean bias with base was −4.2 μg S m^−3^, and mean bias with Base_Het was −1.3 μgS m^−3^) (Fig. 7). Despite the overall improvement in model performance in the Base_Het simulation, a substantial gap in modeled and measured ${\text{PM}}_{2.5,\text{sulf}}$ still existed on 22 December. Daily averaged modeled ${\text{SO}}_{2}$, ${\text{NO}}_{2}$, HCHO, and TMI concentrations (from base HCMAQ, representing a lower bound for ${\text{SO}}_{2}$ consumption) for this time period show that peak ${\text{PM}}_{2.5,\text{sulf}}$ concentrations coincide with the co-occurrence of heightened ${\text{SO}}_{2}+\text{TMI}+{\text{NO}}_{2}$ concentrations (Fig. S10). On 22 December, while ${\text{SO}}_{2}$ concentrations reached a daily average of ~22 ppb, ${\text{NO}}_{2}$ and TMI concentrations were ~1/2 of the concentrations they were on 7–8 December.

The enhancement in ${\text{PM}}_{2.5,\text{sulf}}$ is mainly attributed to ${\text{SO}}_{4}^{2-}$ increases, as HMS maximum daily concentrations over this grid cell for this time period were ~0.002 μg S m^−3^ for Base_Het and the three sensitivity simulations. Simulated HMS for Base_Het and the sensitivities for the winter 2016 case were also ~0.002 μg S m^−3^ (~0.006 μgm^−3^). While the HCMAQ estimates may be low, they are within the range of daily filter measurements of a polluted episode (from ~0 to 1.2 μg m^−3^) reported in Ma et al. (2020).

Normalized mean biases (NMBs) for ${\text{PM}}_{2.5,\text{sulf}}$ in the base simulations over the Northern Hemisphere ranged between −90% and 800%, and NMB for the Base_Het simulation ranged from −90% to 980% (Fig. S11). The largest positive NMB occurred at a site in the western US for both simulations and is due to very low modeled and observed concentrations. The mean biases at this location were only ~0.1–0.2 μg S m^−3^ for both simulations, as this region was generally not affected by our updates (Fig. 6). Improvement of negative NMB occurred in the Base_Het run in some parts of the eastern US and Canada; however it caused and/or increased NMB in the positive direction in most of the eastern US and Canada (Fig. S11). The change in NMB in Europe was not significant, despite enhancements from HMS formation. Regionally aggregated model performance metrics can be found in Table S1.

### 3.3 CMAQ model performance changes over the Contiguous United States with the implementation of heterogeneous sulfur chemistry

**Figure 8.**
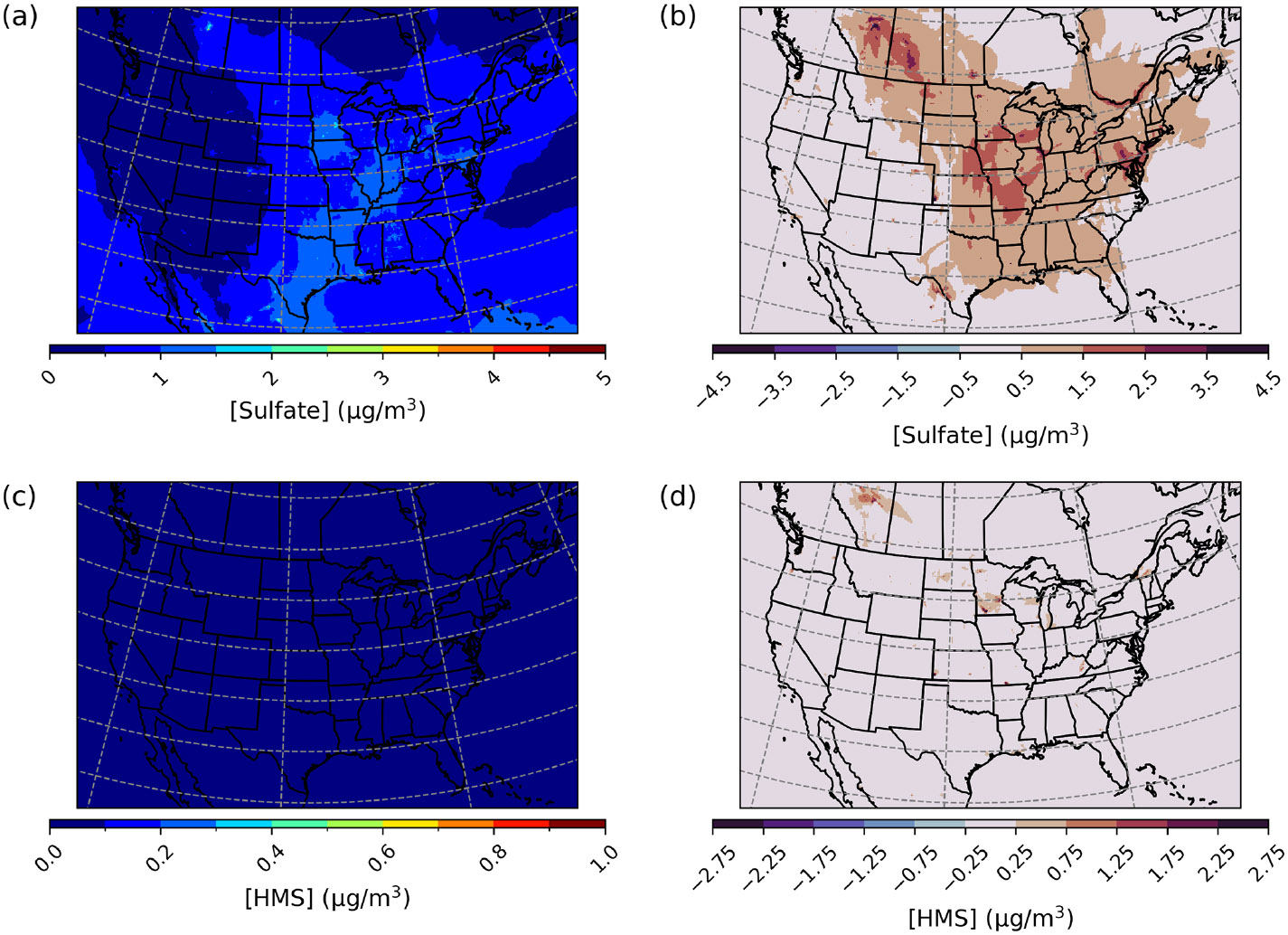
Episode average sulfate **(a)** and HMS **(c)** concentrations in the base simulation along with maximum daily differences in sulfate **(b)** and HMS **(d)** concentrations between the Base_Het and base CMAQ simulations over the Contiguous United States for a wintertime episode (January 2016). HMS formation was not included in base CMAQ, and HMS mass concentrations are multiplied by the ratio of sulfate to HMS molecular mass.

CMAQ was run over the CONUS domain for both a winter- and a summertime episode to ensure that heterogeneous sulfur chemistry updates intended to be significant during dark and cold episodes had minimal impact on domains and episodes where this chemistry is less likely to dominate. For a January 2016 simulation over the CONUS domain, ${\text{PM}}_{2.5,\text{sulf}}$ enhancements can mainly be seen over the eastern US and western Canada (Fig. 8) and are on average enhanced by up to ~1.5 μgm^−3^. Average daily enhancements in Base_Het ${\text{SO}}_{4}^{2-}$ for the entire episode were up to ~1.5 μg m^−3^ as well, and daily average Base_Het HMS enhancements were up to ~0.7 μgm^−3^.

However, ${\text{PM}}_{2.5,\text{sulf}}$ maximum daily enhancements for this episode reached 28 μg m^−3^ at a grid cell in southwestern Kansas (37.3983° N, 101.9184° W; grid cell 121, 177). It should be noted that total modeled daily averaged PM_2.5_ concentrations at this grid cell were 575 μg m^−3^, signifying a major PM event. ${\text{PM}}_{2.5,\text{sulf}}$ enhancements from ${\text{SO}}_{4}^{2-}$ at this grid cell and on this day contributed ~75%, with HMS contributing ~25%. However, the leading ${\text{PM}}_{2.5,\text{sulf}}$ formation pathway at this grid cell in the Base_Het run was the TMI-catalyzed $O_{2}$ oxidation pathway in ALW (Fig. S12). This pathway dominates at a few other locations (Fig. S12); however, gas-phase oxidation of ${\text{SO}}_{2}$ by OH is the leading secondary ${\text{PM}}_{2.5,\text{sulf}}$ formation pathway spatially, followed by cloud–aqueous oxidation by $H_{2}O_{2}$ and ${\text{HNO}}_{4}$.

The maximum daily average enhancement in HMS was ~13 μg m^−3^ and occurred in the Ozarks in south-central Missouri (36.6721° N, 92.9428° W; grid cell 114, 243), also coinciding with a major PM event (model daily average PM_2.5_ concentrations of 301 μg m^−3^) (Fig. 8).

${\text{PM}}_{2.5,\text{sulf}}$ concentration enhancements in the TMI_ sens simulation are similar to those in Base_Het, with a lower contribution from ${\text{SO}}_{4}^{2-}$ production and a higher contribution from HMS production (Fig. S12a and b). The highest maximum daily enhancement for the TMI_sens simulation occurred in the same grid cell in southwestern Kansas discussed previously and was slightly lower than the Base_Het simulation’s maximum daily average enhancement (27.7 μg m^−3^). However, the percentage of ${\text{PM}}_{2.5,\text{sulf}}$ that was ${\text{SO}}_{4}^{2-}$ and HMS was ~10% (3.2μgm^−3^) and ~90% (28.3 μg m^−3^ or 24.5 μg m^−3^ ${\text{SO}}_{4}$ eq.). Other instances of HMS concentrations higher than 5 μg m^−3^ for this run were infrequent and coincided with major PM_2.5_ events (PM_2.5_ concentrations > 100 μg m^−3^). The highest maximum daily enhancements in ${\text{PM}}_{2.5,\text{sulf}}$ for the $\text{TMI}\_{\text{NO}}_{2}\_\text{sens}$ and All_Ionic runs were 22 and 18 μg m^−3^ respectively and occurred at the same grid cell in southwestern Kansas. Enhancements in ${\text{PM}}_{2.5,\text{sulf}}$ in this grid cell were mainly attributed to HMS for both the $\text{TMI}\_{\text{NO}}_{2}\_\text{sens}$ and the All_Ionic simulations and were ~14–15 μg m^−3^, further demonstrating the importance of HMS to total ${\text{PM}}_{2.5,\text{sulf}}$ when a temperature- and pH-dependent TMI-catalyzed $O_{2}\;k_{\text{chem}}$ is used during a cold major PM event.

**Figure 9.**
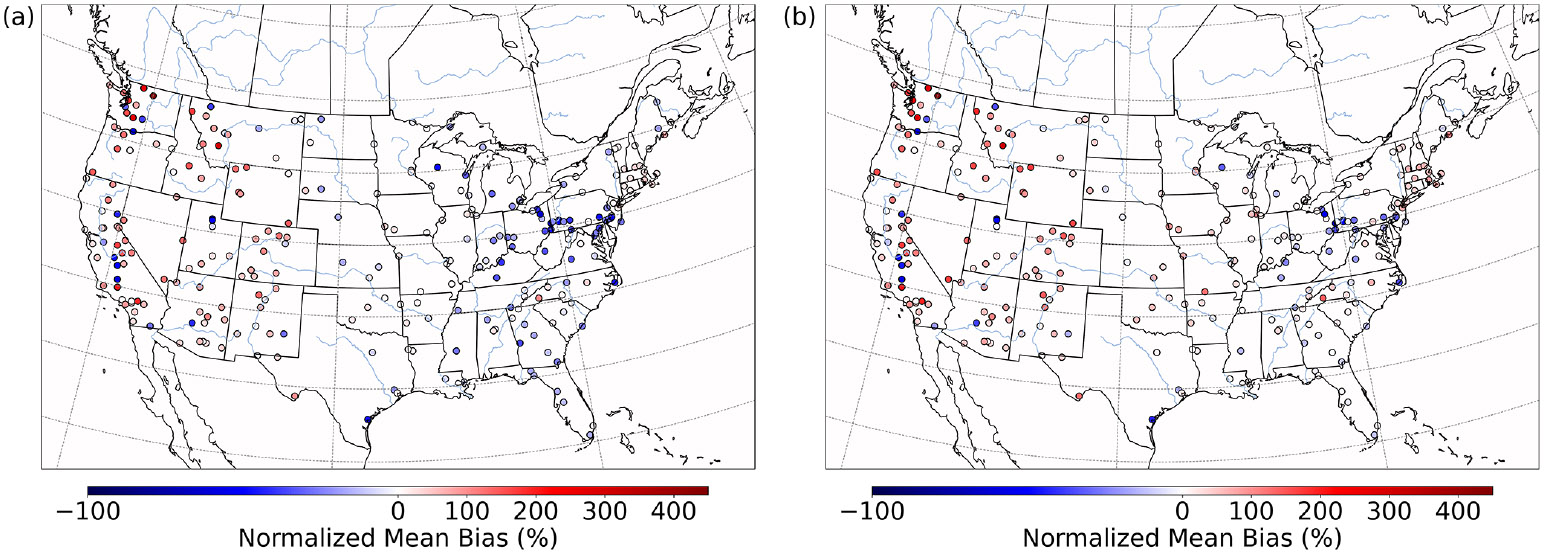
Normalized mean bias in ${\text{PM}}_{2.5,\text{sulf}}$ concentrations by monitor over the CONUS domain for a wintertime episode (January 2016) for base CMAQ **(a)** and Base_Het CMAQ **(b)**.

Both the base and the Base_Het CONUS simulations overestimate measurements in the western US (Fig. 9), with the highest positive NMB being ~450% in Washington state with little difference in the NMB between the two runs – as this region was generally not affected by the implementation of heterogeneous sulfur chemistry (Fig. 8). It should also be noted that mean bias in the western US is fairly low (Fig. S13), and therefore the high NMB in this region is a result of overall low ${\text{PM}}_{2.5,\text{sulf}}$ concentrations (both modeled and measured). Negative NMB of ${\text{PM}}_{2.5,\text{sulf}}$ in the eastern US in the base run are ameliorated in the Base_Het runs, particularly in the Ohio River valley and southeastern US; however, areas in the east with good model ${\text{PM}}_{2.5,\text{sulf}}$ performance in the base run were mostly overpredicted in the Base_Het run. Model performance differences across the additional sensitivity runs were minimal; however, all sensitivity runs had a lower positive NMB at the AQS sites around Chicago compared to Base_Het (Fig. S14), and the TMI_sens simulation had the lowest positive NMB and mean bias at the AQS site in south-central Missouri out of all the newly implemented model runs.

**Figure 10.**
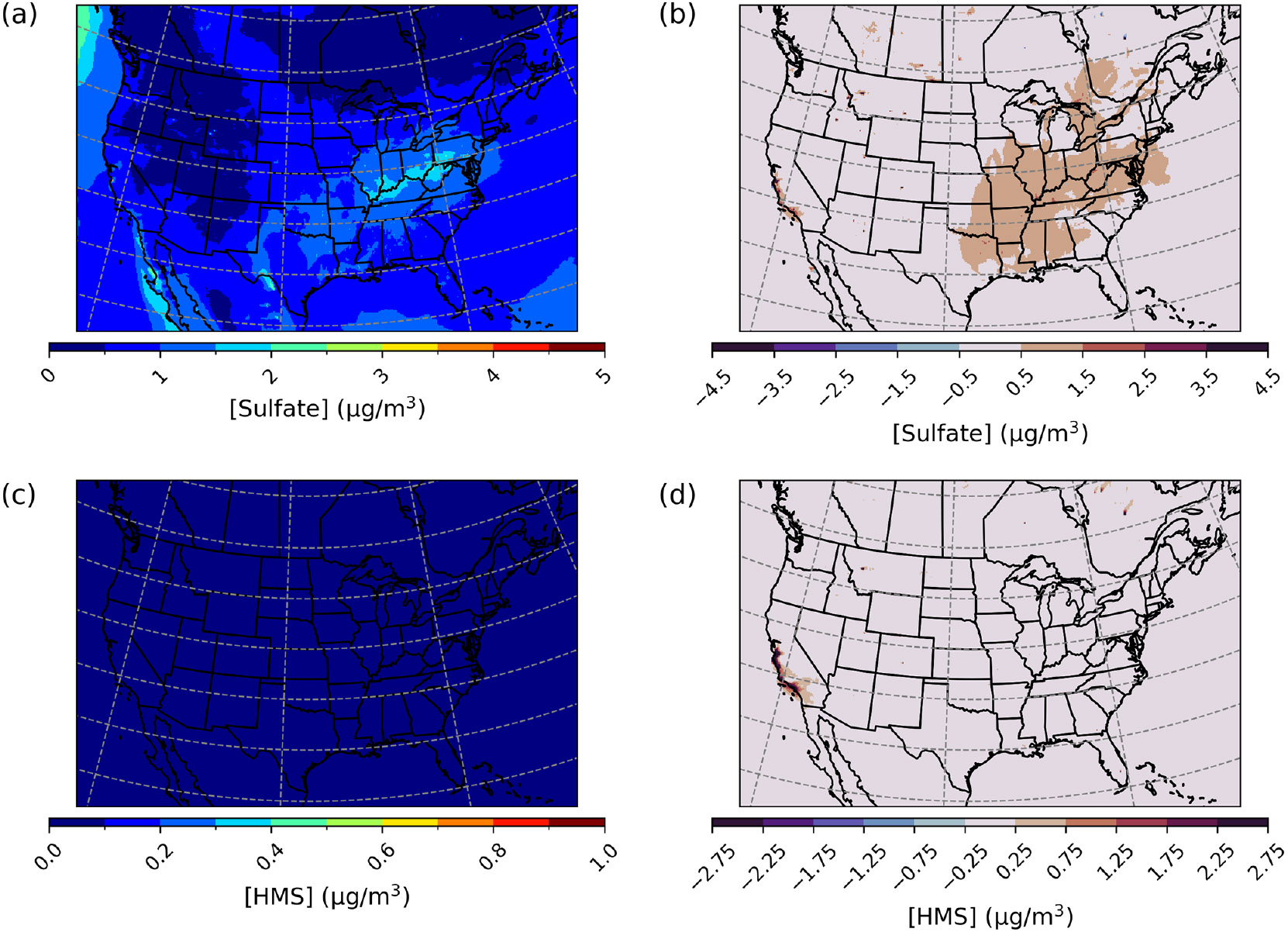
Episode average sulfate **(a)** and HMS **(c)** concentrations in the base simulation along with maximum daily differences in sulfate **(b)** and HMS **(d)** concentrations between the Base_Het and base CMAQ simulations over the Contiguous United States for a summertime episode (July 2016). HMS formation was not included in base CMAQ, and HMS mass concentrations are multiplied by the ratio of sulfate to HMS molecular mass.

For a July 2016 simulated episode over the CONUS domain, overall daily enhancements in Base_Het ${\text{PM}}_{2.5,\text{sulf}}$ were on average 0.05 μg m^−3^ across the entire domain. Daily ${\text{SO}}_{4}^{2-}$ enhancements were more prevalent spatially over the eastern part of the US, particularly in the south and Ohio River valley (Fig. 10), and HMS formation was more prevalent in the western part of the domain.

Maximum daily ${\text{PM}}_{2.5,\text{sulf}}$ enhancements in the Base_Het model run reached up to 789 μgm^−3^, at a grid cell in Monterey County, California (36.2839° N, 121.9208° W; grid cell 135, 30), on 26 July. This was largely due to high daily averaged HMS concentrations (~533 μgm^−3^) (Fig. 10) and coincided with the Soberanes fire at this location in late July (Queally, 2016). ${\text{SO}}_{4}^{2-}$ concentration enhancements were also significant at this grid cell (~255 μg m^−3^). It should be noted that modeled daily averaged PM_2.5_ and HCHO concentrations for this day and grid cell were also extremely high (9389 μg m^−3^ and 577 ppbv respectively).

In the same grid cell and at the same time as the occurrence of maximum daily enhancements in ${\text{PM}}_{2.5,\text{sulf}}$ in the Base_Het run, TMI_sens-simulated ${\text{PM}}_{2.5,\text{sulf}}$ increased by ~791 μgm^−3^, with HMS contributing ~732 μgm^−3^. Similar to results in other domains and episodes, in the TMI_sens simulation, HMS formation is higher, and ${\text{SO}}_{4}^{2-}$ enhancement decreases in comparison to Base_Het. Average daily ${\text{SO}}_{4}^{2-}$ enhancements in TMI_sens reached up to 4 μg m^−3^ and in Base_Het reached up to 11 μgm^−3^, and both occurred in the same grid cell in Monterey, California, mentioned before. For the $\text{TMI}\_{\text{NO}}_{2}\_\text{sens}$ simulation, maximum daily enhancements in ${\text{PM}}_{2.5,\text{sulf}}$ concentrations are similar to TMI_sens (~791 μg m^−3^), with a slightly lower contribution from HMS (~720 μg m^−3^). Maximum daily ${\text{PM}}_{2.5,\text{sulf}}$ enhancements were the lowest in the All_Ionic model run but were still a substantial enhancement (~788 μgm^−3^; occurring in the same grid cell and at the same time as all other model run maximum daily enhancements), with HMS contributing ~709 μg m^−3^ and ${\text{SO}}_{4}^{2-}$ contributing ~79 μg m^−3^ to this enhancement. The spatial distributions of ${\text{SO}}_{4}^{2-}$ enhancements for all sensitivity runs were similar to those of the Base_Het ${\text{SO}}_{4}^{2-}$ enhancements (Fig. S15).

**Figure 11.**
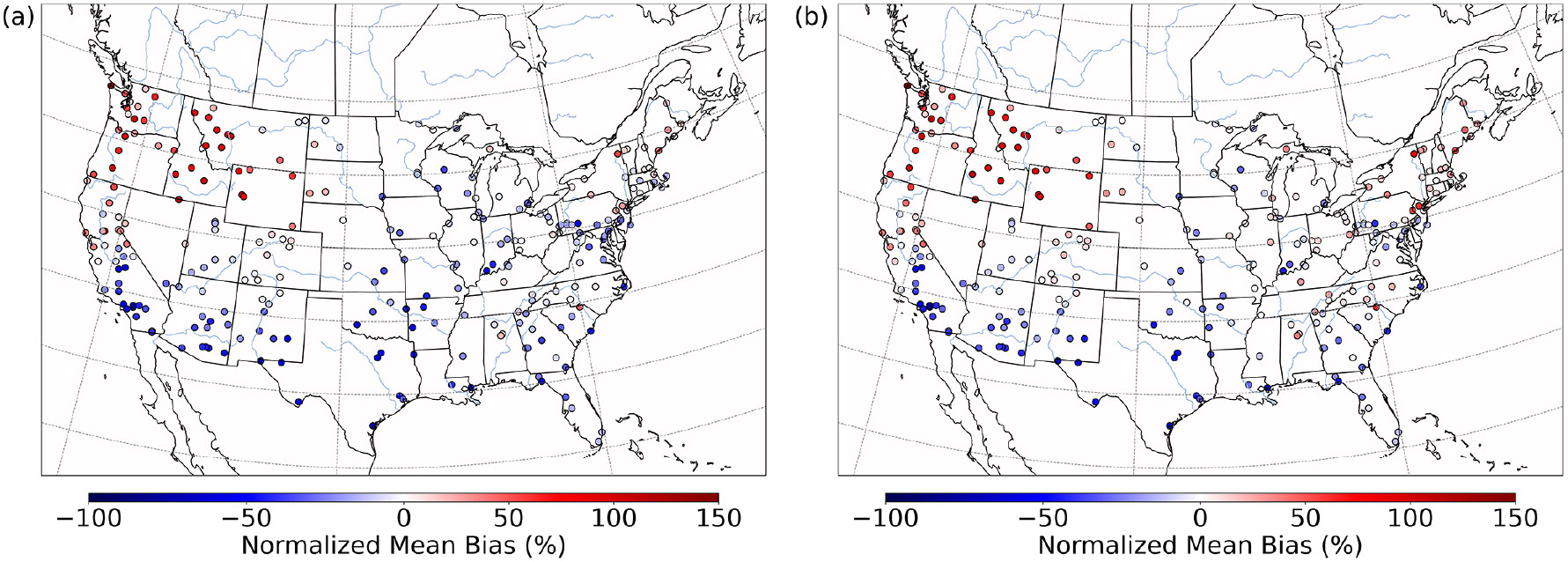
Normalized mean bias in ${\text{PM}}_{2.5,\text{sulf}}$ concentrations by monitor over the CONUS domain for a summertime episode (July 2016) for base CMAQ **(a)** and Base_Het CMAQ **(b)**.

Both the base and the Base_het simulations mainly overestimate ${\text{PM}}_{2.5,\text{sulf}}$ concentrations in the northwestern US and have similar performance (Figs. 11 and S16) due to this region generally not being affected by heterogeneous sulfur chemistry updates (with the exception of a few locations that are not in the same grid cells as an AQS monitor). Although there were significant enhancements in HMS and off the Californian coast, HMS concentrations greater than 5 μg m^−3^ were limited to a few grid cells on 26 July (when maximum concentrations of HMS were predicted) and did not reach the nearest AQS monitors. Higher daily HMS concentrations were predicted south of Monterey down the Californian coast the following 2 d (which did not have a corresponding AQS measurement). Increased ${\text{SO}}_{4}^{2-}$ formation in the Base_Het simulation improved modeled underestimates in some parts of the eastern US (Figs. 11 and S16), particularly in parts of the southeast (Fig. S17).

Although the implementation of heterogeneous sulfur chemistry generally led to minimal enhancements in both ${\text{SO}}_{4}^{2-}$ and HMS over the entire CONUS domain for both episodes, it did slightly increase ${\text{SO}}_{4}^{2-}$ in the eastern US and substantially increased HMS concentrations during major PM events, including but not limited to wildfires. Enhancements in ${\text{SO}}_{4}^{2-}$ could potentially impact the formation of secondary organic aerosol (SOA; Fan et al., 2022). ${\text{SO}}_{4}^{2-}$ is an important nucleophile in the formation of isoprene and epoxydiol (IEPOX)-derived organosulfates (Surratt et al., 2010), and enhancements in ${\text{SO}}_{4}^{2-}$ concentration may help reduce model underpredictions for IEPOX organosulfates (Budisulistiorini et al., 2017). Aqueous ${\text{SO}}_{4}^{2-}$ in the presence of ${\text{Fe}}^{3+}$ and other isoprene-derived SOA precursors has been shown to enhance the production of C2–C4 organosulfur compounds as well (Huang et al., 2019). Modeled enhancements in both ${\text{SO}}_{4}^{2-}$ and HMS during wildfires are both particularly high due to extraordinarily high emissions during these events in general. TMI_sens predicted the highest HMS concentrations across all model simulations; however, max daily ${\text{SO}}_{4}^{2-}$ concentrations in $\text{TMI}\_{\text{NO}}_{2}\_\text{sens}$ were higher in comparison to TMI_sens, indicating the importance of the heterogeneous ${\text{NO}}_{2}$ oxidation pathway during wildfire events. Wildfires release high concentrations of black carbon, which can inhibit modeled photolysis of ${\text{NO}}_{2,}$, and high in-plume oxidant concentrations can facilitate its regeneration (Buysse et al., 2019; Xing et al., 2017).

## 4 Discussion

### 4.1 Improved model performance with heterogeneous sulfur chemistry in dark, cold episodes

Traditional mechanisms of secondary sulfate formation have not been able to reproduce the high observed sulfate concentrations experienced during Fairbanks and North Pole, AK, winters. The added heterogeneous sulfur chemistry in ALW in this study enhanced modeled wintertime ${\text{PM}}_{2.5,\text{sulf}}$ concentrations in this domain and in China, where high wintertime ${\text{PM}}_{2.5,\text{sulf}}$ concentrations have also been observed (ADEC, 2017; Cheng et al., 2016; Ma et al., 2020; Moch et al., 2018; Wang et al., 2016; Zhang et al., 2021a). Inclusion of these reactions improved model performance in these regions (Figs. 5 and 7). ${\text{PM}}_{2.5,\text{sulf}}$ enhancements between the base and Base_Het simulations during a PM episode (E1) in the Fairbanks domain showed that heterogeneous sulfur chemistry in ALW can increase daily ${\text{PM}}_{2.5,\text{sulf}}$ concentrations by up to 11 μg m^−3^ across the entire domain for this dark and cold modeling episode (Fig. 2). At grid cells in downtown Fairbanks and North Pole, maximum daily enhancements in ${\text{PM}}_{2.5,\text{sulf}}$ during E1 can range between ~1–1.5 and ~4–25 μg m^−3^ respectively (Figs. 2 and S3) across all of the heterogeneous sulfur chemistry configurations tested in this work. In this same domain during E2, modeled secondary ${\text{PM}}_{2.5,\text{sulf}}$ formation takes place primarily via in-cloud formation pathways (Figs. 4 and S7), highlighting the enduring importance of cloud–aqueous chemistry in modeling ${\text{PM}}_{2.5,\text{sulf}}$ formation when substantial liquid cloud water is present.

${\text{PM}}_{2.5,\text{sulf}}$ enhancements from heterogeneous sulfur chemistry for the wintertime HCMAQ simulations occurred mostly in China, South Asia, and Europe (Fig. 6). Maximum daily enhancements in ${\text{PM}}_{2.5,\text{sulf}}$ ranged between 33– 54 μgS m^−3^ across heterogeneous sulfur chemistry configurations tested in this work and were all located in China. Heterogeneous sulfur chemistry updates also improved negative model bias for ${\text{PM}}_{2.5,\text{sulf}}$ in Canada (Table S1), where wintertime temperatures can be extremely cold and residential home heating along with industrial emissions may be high (Cho et al., 2009; Liggio et al., 2016; Stroud, 2009).

In general, there were minimal changes in predicted ${\text{PM}}_{2.5,\text{sulf}}$ in regions and during episodes that do not experience extremely cold and dark conditions. Positive model bias in estimating ${\text{PM}}_{2.5,\text{sulf}}$ in Europe for the HCMAQ simulation increases slightly in the Base_Het, TMI_sens, and $\text{TMI}\_{\text{NO}}_{2}\_\text{sens}$ simulations but remains unchanged in the All_Ionic simulation. Similarly, model bias in estimating ${\text{PM}}_{2.5,\text{sulf}}$ in the US for HCMAQ also increases slightly from the base in all the newly implemented model runs by 0.05 μg m^−3^ (Table S1).

The inclusion of heterogeneous sulfur chemistry had a smaller impact on modeled ${\text{PM}}_{2.5,\text{sulf}}$ over the CONUS domain (in comparison to the Fairbanks and HCMAQ domains), with the exception of high-PM events. Interestingly, the inclusion of these missing pathways reduced some of the negative bias in ${\text{PM}}_{2.5,\text{sulf}}$ concentrations in the southeast during warm and light summertime conditions (Figs. 11, S16, and S17), and this bias reduction is mostly due to ${\text{SO}}_{4}^{2-}$ enhancements (Figs. 10 and S15). ${\text{SO}}_{4}^{2-}$ can participate as a reactant and a modulator of pH in the heterogeneous formation of IEPOX-derived organosulfates (Marais et al., 2016; Pye et al., 2017; Pye et al., 2020), and improved ${\text{SO}}_{4}^{2-}$ negative bias may also improve model performance of IEPOX SOAs.

### 4.2 Contribution of HMS to ${\text{PM}}_{2.5,\text{sulf}}$

In this study we found that HMS, a previously untracked aerosol species in CMAQ, can contribute substantially to total ${\text{PM}}_{2.5,\text{sulf}}$, depending on the HCHO and ${\text{SO}}_{2}$ concentrations, temperature, and heterogeneous sulfur chemical kinetics chosen. During E1, HMS concentrations in North Pole are higher in comparison to Fairbanks. Given the stagnant conditions for this domain and episode, emissions tend to stay local (ADEC, 2017; Gaudet et al., 2012; Tran and Molders, 2011). Thus, HCHO from residential wood combustion and ${\text{SO}}_{2}$ from home-heating oil in North Pole, a largely residential area, compared to downtown Fairbanks are the likely dominating reasons for higher HMS concentrations when comparing the two areas.

Modeled HMS generally had a smaller impact on both the northern hemispheric and the CONUS domains (generally contributing < 1 μg m^−3^), with a few anomalous exceptions. Modeled HMS in HCMAQ runs primarily appeared in Europe and China, where wintertime HMS has previously been predicted (Moch et al., 2020). In the CONUS domain, HMS concentrations were predicted to be high during major PM events (including but not limited to wildfires), which illuminates the importance of HMS during high atmospheric loadings of both ${\text{SO}}_{2}$ and HCHO.

Across all modeled domains and episodes, the TMI_sens model run predicted the highest HMS concentrations and may represent an upper bound on modeled HMS concentrations. Ultimately, more resolved measurements of speciated PM_2.5_ that can separate HMS and ${\text{SO}}_{4}^{2-}$ (Campbell et al., 2022) can help discern their relative contributions to ${\text{PM}}_{2.5,\text{sulf}}$ mass and help constrain future modeled heterogeneous sulfur chemical kinetics.

### 4.3 ${\text{PM}}_{2.5,\text{sulf}}$ formation pathways of interest during cold and dark episodes

In addition to the inclusion of both heterogeneous ${\text{SO}}_{4}^{2-}$ and both in-cloud and heterogeneous HMS formation in CMAQ, we determined which ${\text{PM}}_{2.5,\text{sulf}}$ formation pathways are the most important given ionic strength, pH, and temperature regimes characteristic of dark and cold conditions. Across both the Fairbanks and the CONUS domains in the Base_Het simulation during wintertime, the most prevailing heterogeneous ${\text{SO}}_{4}^{2-}$ formation pathway was the TMI-catalyzed $O_{2}$ pathway (Figs. 2, 4, S12). In the TMI_sens E1 in Fairbanks, however, this formation pathway was the third-most important, behind HMS formation and the ${\text{NO}}_{2}$ pathway (Fig. S3). Although the modeled pH for TMI_sens ranged between 3–6 for Fairbanks and North Pole and for both episodes (Fig. S4), which included the optimal pH for this pathway (pH = 4.2; Ibusuki and Takeuchi, 1987), the dampening of this pathway can also be attributed to the extremely cold temperatures (modeled average −30 °C or 243 °K).

TMI_sens-modeled aerosol pH was seen to be the least acidic in comparison to all of the other model simulations, especially in North Pole (Fig. S4). As noted before, HMS was the largest contributor to secondary ${\text{PM}}_{2.5,\text{sulf}}$ formation in North Pole, the formation (and loss) rates of which increased with increasing pH (Ervens et al., 2003; Kok et al., 1986) (Fig. 2). Aerosol pH and ALW calculations in ISORROPIA II only consider inorganic species. Organic species (e.g., organic acids) may also increase aerosol acidity (Zuend and Seinfeld, 2012; Zuend et al., 2011), and therefore the predicted aerosol pH in TMI_sens might represent an overprediction. Aerosol pH for the Base_Het, $\text{TMI}\_{\text{NO}}_{2}\_\text{sens}$, and All_Ionic model simulations was similar at both North Pole and Fairbanks, with both sensitivity simulations predicting slightly higher pH than the Base_Het simulation during E1 and slightly lower pH during E2.

The impacts of increased ionic strength were explored with respect to the ${\text{NO}}_{2}$, $O_{3}$, and $H_{2}O_{2}$ oxidation pathways (note that ionic strength inhibition of the $\text{TMI}-O_{2}$ pathway is included in the Base_Het simulations as well). Ionic strength impacts added to the ${\text{NO}}_{2}$ pathway (in $\text{TMI}\_{\text{NO}}_{2}\_\text{sens}$) had the highest impact on the formation of ${\text{SO}}_{4}^{2-}$ via this pathway (Figs. S3 and S7c and d) in the Fairbanks domain for both episodes. Although the ionic strength for this pathway was bounded at 1.14 M, an increase in aerosol ionic strength from 0.1 M to the upper bound of 1.14 M increased $k_{\text{chem}}$ for this pathway by ~2 orders of magnitude (Chen et al., 2019). Ionic strength impacts on the $H_{2}O_{2}$ and $O_{3}$ heterogeneous sulfur oxidation pathways had minimal impact during the wintertime PM episodes in Fairbanks and North Pole, only accounting for ≤ ~0.2 μg m^−3^ in the All_Ionic model simulation (Figs. S3 and S7). These particular pathways were not assumed to be prolific given the lack of photochemistry during this episode; however with an ionic strength change from 0 to 5 M, the third-order aqueous-phase rate coefficient for the $H_{2}O_{2}$ heterogeneous sulfur pathway can increase by more than 40% regardless of pH or temperature (Maaß et al., 1999; Millero et al., 1989). The ionic strength used to calculate the ionic strength effect factor for this $k_{\text{chem}}$ was limited to a maximum of 5 M; however recent studies have observed significant ionic strength enhancement up to 14 M (Liu et al., 2020). For ${\text{SO}}_{4}^{2-}$ formation via heterogeneous oxidation by $O_{3}$, $k_{\text{chem}}$ for this pathway can increase by ~80%, with an ionic strength increase from 0.1 to 0.8 M (Lagrange et al., 1994; Song et al., 2021a) at temperatures characteristic of Fairbanks winters; however these effects were not seen due to the lack of ozone modeled given the dark and cold conditions.

## 5 Conclusion

Air quality modeling of secondary sulfate has traditionally only included in-cloud aqueous- and gas-phase ${\text{SO}}_{2}$ oxidation pathways, often resulting in underpredictions of observed ${\text{PM}}_{2.5,\text{sulf}}$, especially during the cold and polluted conditions characteristic of wintertime PM and haze events (ADEC, 2017; Gao et al., 2016). In this study, we implemented heterogeneous sulfur chemistry in aerosol liquid water in CMAQ to resolve model–measurement gaps in ${\text{PM}}_{2.5,\text{sulf}}$ concentrations during extreme wintertime PM episodes in and around Fairbanks, Alaska. We compared modeled ${\text{PM}}_{2.5,\text{sulf}}$ (${\text{SO}}_{4}^{2-}+\text{HMS}$) concentrations to sulfate measurements at several measurement sites (under the assumption that HMS may be included in the sulfate observations, Dovrou et al., 2019). Negative model bias improved in Fairbanks during winter and decreased with these updates and also improved in Beijing, another location known to experience wintertime haze events. When applied more broadly to larger domains and other seasons, the update also resolved underestimations of ${\text{PM}}_{2.5,\text{sulf}}$ concentrations in both the United States and globally; however, it did not have a huge impact when applied over domains that were not as dark and cold. HMS was found to be an important contributor to ${\text{PM}}_{2.5,\text{sulf}}$ mass during dark and cold episodes; however, to better understand the ratio of sulfate to HMS, more observations of HMS are necessary. Recently, the Alaskan Layered Pollution and Chemical Analysis (ALPACA) field campaign (Simpson et al., 2024) was conducted in and around Fairbanks during January–February 2022 and offers observations to elucidate important sulfate and HMS formation pathways in the area and better characterize source apportionment of ${\text{PM}}_{2.5,\text{sulf}}$. Ultimately, HMS and sulfate formed via the TMI-catalyzed $O_{2}$ and ${\text{NO}}_{2}$ pathways proved to be the most important to ${\text{PM}}_{2.5,\text{sulf}}$ formation pathways in Fairbanks and North Pole and require further investigation in the context of ${\text{PM}}_{2.5,\text{sulf}}$ control strategies. Finally, while this study aims to include ionic strength, pH, and temperature impacts on ${\text{PM}}_{2.5,\text{sulf}}$ formation in CMAQ, the ionic strength, pH, and temperature ranges under which Henry’s law, reaction rates, and other coefficients and parameters were derived experimentally may not be representative of concentrated aerosol water or that of extremely cold and dark wintertime conditions. Laboratory studies are needed to extend the bounds of these parameters and determine rate expressions appropriate for the concentrated conditions characteristic of aerosol water.

## Supplementary Material

Supplement1

## Figures and Tables

**Table 1.** Pseudo-first-order rate constants ($k_{\text{chem}}$) and ionic strength (I) factors for each simulation.

Uptake gas	Model simulation	$k_{\text{chem}}(s^{-1})$	Product	Reference
$O_{3}$	$\text{Base}\_\text{Het},\text{TMI}\_\text{sens},\text{TMI}\_{\text{NO}}_{2}\_\text{sens}$	$k_{O_{3}}=k_{1}[H_{2}{\text{SO}}_{3}]+k_{2}[{\text{HSO}}_{3}^{-}]+k_{3}[{\text{SO}}_{3}^{2-}]$ $k_{1}=2.4\times 10^{4}\;M^{-1}s^{-1}$ $k_{2}=3.7\times 10^{5}\times e^{-5530\times (\frac{1}{T}-\frac{1}{298})}\;M^{-1}s^{-1}$ $k_{3}=1.5\times 10^{9}\times e^{-5280\times (\frac{1}{T}-\frac{1}{298})}\;M^{-1}s^{-1}$	${\text{SO}}_{4}^{2-}$	Hoffmann and Calvert (1985)
	All_Ionic	$k_{O_{3},I}=k_{O_{3}}\times (1+(b\times I_{O_{3}}))$ $I_{O_{3},\text{max}}=1.2M$ b=1.94 ^a^	${\text{SO}}_{4}^{2-}$	Lagrange et al. (1994)
$H_{2}O_{2}$	$\text{Base}\_\text{Het},\text{TMI}\_\text{sens},\text{TMI}\_{\text{NO}}_{2}\_\text{sens}$	$k_{H_{2}O_{2}}=\frac{k_{4}[H^{+}][{\text{HSO}}_{3}^{-}]}{1+K[H^{+}]}$ $k_{4}=7.45\times 10^{7}\times e^{-4430\times (\frac{1}{T}-\frac{1}{298})}\;M^{-1}s^{-1}$ $K=13M^{-1}$	${\text{SO}}_{4}^{2-}$	McArdle and Hoffmann (1983)
	All_Ionic	$k_{H_{2}O_{2},I}=k_{4}\times [{\text{HSO}}_{3}^{-}]\times [H^{+}]\times 10^{0.36I_{H_{2}O_{2}}-\frac{1.018\sqrt{I_{H_{2}O_{2}}}}{1+0.17\sqrt{I_{H_{2}O_{2}}}}}$ $k_{4}=9.1\times 10^{7}\times e^{-3572\times (\frac{1}{T}-\frac{1}{298})}\;M^{-1}s^{-1}$ $I_{H_{2}O_{2},\text{max}}=5M$	${\text{SO}}_{4}^{2-}$	Maaß et al., 1999
${\text{NO}}_{2}$	Base_Het, TMI_sens	$k_{{\text{NO}}_{2}}=k_{5}[S(\text{IV})]$ ^b^ $k_{5}=2\times 10^{6}\;M^{-1}s^{-1}$	${\text{SO}}_{4}^{2-}$	Lee and Schwartz (1983b)
	$\text{TMI}\_{\text{NO}}_{2}\_\text{sens},{\text{All}}_{\_}\text{Ionic}$	$k_{{\text{NO}}_{2},I}=k_{5,\text{ionic}}[S(\text{IV})]$ ^b^ $k_{5,\text{ionic}}=10^{6.1+\frac{3.1\times I_{{\text{NO}}_{2}}}{I_{{\text{NO}}_{2}}+0.2}}$ $I_{{\text{NO}}_{2},\text{max}}=1.14M$	${\text{SO}}_{4}^{2-}$	Chen et al. (2019)
${\text{SO}}_{2}$	Base_Het	$k_{{\text{TMIO}}_{2},I}=\frac{k_{6}[\text{Mn}(\text{II})]+k_{7}[\text{Fe}(\text{III})]+k_{8}[\text{Mn}(\text{II})][\text{Fe}(\text{III})]}{1+75\times S{\left(\text{VI}\right)}_{S}^{0.67}}\times 10^{b\times \frac{\sqrt{I_{\text{TMI}}}}{1+\sqrt{I_{\text{TMI}}}}}$ $k_{6}=750\;M^{-1}s^{-1}$ $k_{7}=2600\;M^{-1}s^{-1}$ $k_{8}=1e^{10}\;M^{-2}s^{-1}$ $I_{\text{TMI},\text{max}}=2M$ b=−4.07 ^c^	${\text{SO}}_{4}^{2-}$	Martin and Good (1991), Martin and Hill (1987)
	$\text{TMI}\_\text{sens},\text{TMI}\_{\text{NO}}_{2}\_\text{sens},\text{All}\_\text{Ionic}$	$k_{{\text{TMIO}}_{2},I}=k_{6}{\left[H^{+}\right]}^{-0.74}[\text{Mn}(\text{II})][\text{Fe}(\text{III})])(\text{for pH}\leq 4.2)$ $k_{6}=3.72\times 10^{7}\times e^{-8431.6\times (\frac{1}{T}-\frac{1}{297})}\times 10^{b\times \frac{\sqrt{I_{\text{TMI}}}}{1+\sqrt{I_{\text{TMI}}}}}\;M^{-2}s^{-1}$ $k_{{\text{TMIO}}_{2},I}=k_{7}{\left[H^{+}\right]}^{0.67}[\text{Mn}(\text{II})][\text{Fe}(\text{III})](\text{for pH}>4.2)$ $k_{7}=2.51\times 10^{13}\times e^{-8431.6\times (\frac{1}{T}-\frac{1}{297})}\times 10^{b\times \frac{\sqrt{I_{\text{TMI}}}}{1+\sqrt{I_{\text{TMI}}}}}\;M^{-2}s^{-1}$ $I_{\text{TMI},\text{max}}=2M$ b=−4.07 ^c^	${\text{SO}}_{4}^{2-}$	Ibusuki and Takeuchi (1987), Martin and Hill (1987)
HCHO^d^	$\text{Base}\_\text{Het},\text{TMI}\_\text{sens},\text{TMI}\_{\text{NO}}_{2}\_\text{sens}\;\text{All}\_\text{Ionic}$	$k_{8}[{\text{HSO}}_{3}^{-}]+k_{9}[{\text{SO}}_{3}^{2-}]$ $k_{8}=790\times e^{-2900\times (\frac{1}{T}-\frac{1}{298})}$ $k_{9}=2.5\times 10^{7}\times e^{-2450\times (\frac{1}{T}-\frac{1}{298})}$	HMS	Boyce and Hoffmann (1984)

a The ionic strength cap chosen corresponds with the activation energy for the rate constant, $k_{1}$, which matches the ionic strength cap, b, for sodium perchlorate (${\text{NaClO}}_{4}$).

b $\left[S(\text{IV})](M)=[{\text{SO}}_{2}\cdot H_{2}O]+[{\text{HSO}}_{3}^{-}]+[{\text{SO}}_{3}^{2-}\right]$

c The b value corresponds to the factor associated with Mn-catalyzed $O_{2}$ oxidation of ${\text{SO}}_{2}$, corresponding to ≥ 10^−4^ M sulfur (Martin and Hill, 1987).

d Also included is the loss of HMS from decomposition and OH oxidation (Song et al., 2021a).

**Table 2.** Henry’s law coefficients used per model simulation.

Species	Model simulation	$H_{A}(M\;{\text{atm}}^{-1})$	Reference
$O_{3}$	$\text{Base}\_\text{Het}\;\text{TMI}\_\text{sens}\;\text{TMI}\_{\text{NO}}_{2}\_\text{sens}$	$H_{O_{3}}^{I_{O_{3}}=0}=0.0114\times e^{2300}\times (\frac{1}{T}-\frac{1}{298})$	Kosak-Channing and Helz (1983)
	All_Ionic	$H_{O_{3}}=e^{\frac{2297}{T}-2.659\times I_{O_{3}}+688\times \frac{I_{O_{3}}}{T}-12.19}$ $I_{O_{3},\text{max}}=0.6\;M$	Kosak-Channing and Helz (1983)
$H_{2}O_{2}$	$\text{Base}\_\text{Het}\;\text{TMI}\_\text{sens}\;\text{TMI}\_{\text{NO}}_{2}\_\text{sens}$	$H_{H_{2}O_{2}}^{I_{H_{2}O_{2}=0}}=1.1\times 10^{5}\times e^{7400\times (\frac{1}{T}-\frac{1}{298})}$	O’Sullivan et al. (1996)
	All_Ionic	$H_{H_{2}O_{2}}^{I_{H_{2}O_{2}=0}}=1.3\times 10^{5}\times e^{7300\times (\frac{1}{T}-\frac{1}{298})}$ $\frac{H_{H_{2}O_{2}}}{H_{H_{2}O_{2}}^{I_{H_{2}O_{2}=0}}}=1-1.414\times 10^{-3}\times I_{H_{2}O_{2}}^{2}+0.121I_{H_{2}O_{2}}$ $I_{H_{2}O_{2},\text{max}}=5\;M$	Seinfeld and Pandis (2016), Aliet al. (2014), Liu et al. (2020)
${\text{NO}}_{2}$	$\text{Base}\_\text{Het},\text{TMI}\_\text{sens}\;\text{TMI}\_{\text{NO}}_{2}\_\text{sens}\;\text{All}\_\text{Ionic}$	$H_{{\text{NO}}_{2}}=0.012\times e^{2500\times (\frac{1}{T}-\frac{1}{298})}$	Chameides (1984)
${\text{SO}}_{2}$	$\text{Base}\_\text{Het}\;\text{TMI}\_\text{sens}\;\text{TMI}\_{\text{NO}}_{2}\_\text{sens}$	$[H_{2}{\text{SO}}_{3}]=H_{{\text{SO}}_{2}}\times p_{{\text{SO}}_{2}}$ $[{\text{HSO}}_{3}^{-}]=K_{a1}\times \left[H_{2}{\text{SO}}_{3}\right]∕[H^{+}]$ $[{\text{SO}}_{3}^{2-}]=K_{a2}\times \left[{\text{HSO}}_{3}^{-}\right]∕[H^{+}]$ $H_{{\text{SO}}_{2}}=1.4\times e^{2900\times (\frac{1}{T}-\frac{1}{298})}$ $K_{a1}=0.013\times e^{1960\times (\frac{1}{T}-\frac{1}{298})}$ $K_{a2}=6.6\times 10^{-8}\times e^{1500\times (\frac{1}{T}-\frac{1}{298})}$	Lide and Frederikse (1995)
	All_Ionic*	$H_{{\text{SO}}_{2}}^{I_{{\text{SO}}_{2}}=0}=1.2\times e^{3100\times (\frac{1}{T}-\frac{1}{298})}$ $\frac{H_{{\text{SO}}_{2}}}{H_{{\text{SO}}_{2}}^{I_{{\text{SO}}_{2}}=0}}=10^{\left((\frac{22.3}{T}-0.0997)\times I_{{\text{SO}}_{2}}\right)}$ $K_{a1}^{I}=K_{a1}\times 10^{0.5\sqrt{I}-0.31I}$ $K_{a2}^{I}=K_{a2}\times 10^{1.052\sqrt{I}-0.36I}$ $I_{{\text{SO}}_{2},\text{max}}=6\;M$	Seinfeld and Pandis (2016), Millero et al. (1989)
HCHO	$\text{Base}\_\text{Het},\text{TMI}\_\text{sens}\;\text{TMI}\_{\text{NO}}_{2}\_\text{sens}\;\text{All}\_\text{Ionic}$	$H_{\text{HCHO}}=\frac{H_{\text{HCHO}}^{*}}{1+K_{\text{HCHO}}^{\text{HYD}}}$ $H_{\text{HCHO}}^{*}=3200\times e^{6800\times (\frac{1}{T}-\frac{1}{298})}$ $K_{\text{HCHO}}^{\text{HYD}}=\frac{0.18\times e^{4030\times (\frac{1}{T}-\frac{1}{298})}\times 55.5\;M}{0.0051}$	Seinfeld and Pandis (2016), Ervens et al. (2003), Staudinger and Roberts (1996)

* Aqueous-phase concentrations of S(IV) are calculated similarly to Base_Het, TMI_sens, and $\text{TMI}\_{\text{NO}}_{2\_\text{sens}}$ but with ionic-strength-dependent equilibrium coefficients.

## Data Availability

Model source code, output, and observations used in this paper can be accessed at https://doi.org/10.23719/1530692 (USEPA, 2024b). AQS sulfate measurements were used for model evaluation for the Fairbanks Domain (USEPA, 2024a), CONUS and northern hemispheric domain (USEPA, 2022). For northern hemispheric model evaluation, Canadian sulfate observations were sourced from the NAPS monitoring network (ECCC, 2022); European sulfate was sourced from the EMEP monitoring network (Tørseth et al., 2012); and sulfate measurements from Tsinghua University in Beijing were sourced from Fengkui Duan, Tao Ma, and Shuping Zhang and are not publicly available but are cited (Ma et al., 2020, Zhang et al., 2021).

## Footnotes

1 The United States Clean Air Act requires EPA to set National Ambient Air Quality Standards (NAAQS) for six pollutants, including fine particulate matter (PM_2:5_), and to periodically review those standards. In 2006, EPA updated the NAAQS for PM_2:5_ concentrations averaged over a 24 h time period. This updated standard requires the calculation of the 98th percentile of daily (24 h) PM_2:5_ concentrations for 3 years and that the average of those three 98th percentile concentrations be at or below a threshold of 35 μg m^−3^. For simplicity, we refer to this as the 2006 24 h PM_2:5_ NAAQS.

## Code availability.

The version of CMAQ used in this paper, CMAQv5.3.2, is archived at https://doi.org/10.5281/zenodo.4081737 (USEPA Office of Research and Development, 2020) and is used for the base model runs. Sensitivity model cases were developed on top of the base model setup with additional coding contributions from Kathleen Fahey, Sarwar Golam, Sara Farrell, and Havala Pye and can be accessed at https://doi.org/10.23719/1530692 (USEPA, 2024b).
