# Supplementary material for: Predicted impacts of heterogeneous chemical pathways on particulate sulfur over Fairbanks (Alaska), the Northern Hemisphere, and the Contiguous United States
Source: Atmos Chem Phys. Author manuscript; Available in PMC 2026 Mar 18. (PMC12181941; doi:10.5194/acp-25-3287-2025)
Supplement: Supplement1 [file NIHMS2069158-supplement-Supplement1.pdf]

Supplement of Atmos. Chem. Phys., 25, 3287–3312, 2025
https://doi.org/10.5194/acp-25-3287-2025-supplement
© Author(s) 2025. CC BY 4.0 License.

Atmospheric Chemistry and Physics
Open Access

Supplement of

Predicted impacts of heterogeneous chemical pathways on particulate sulfur over Fairbanks (Alaska), the Northern Hemisphere, and the Contiguous United States

Sara L. Farrell et al.

Correspondence to: Sara L. Farrell (farrell.sara@epa.gov) and Kathleen Fahey (fahey.kathleen@epa.gov)

The copyright of individual parts of the supplement might differ from the article licence.

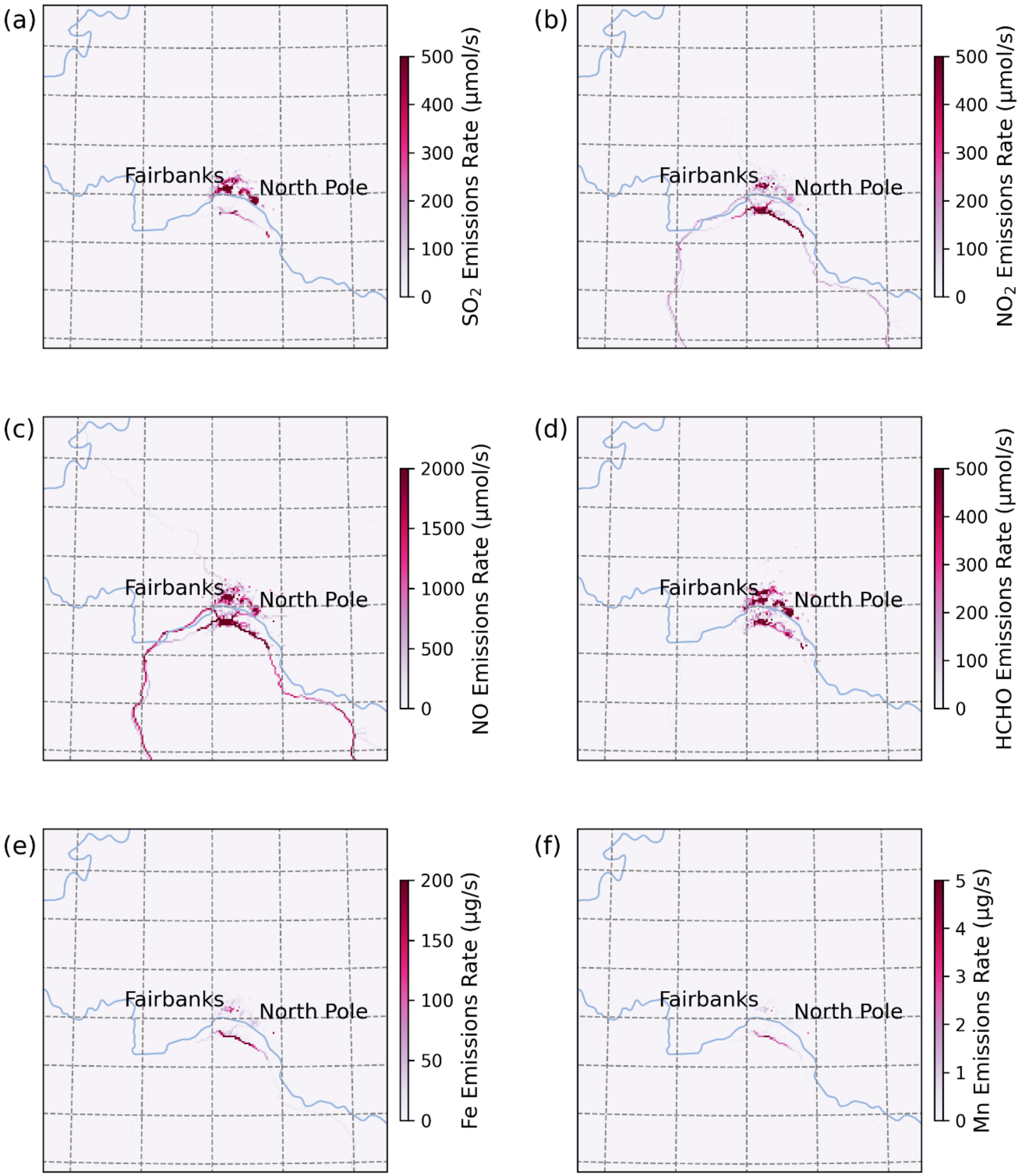

Figure S1. Episode averaged emissions of particulate sulfur precursors for episode 1 (January 25th – February 11th,2008) in the Fairbanks Domain.

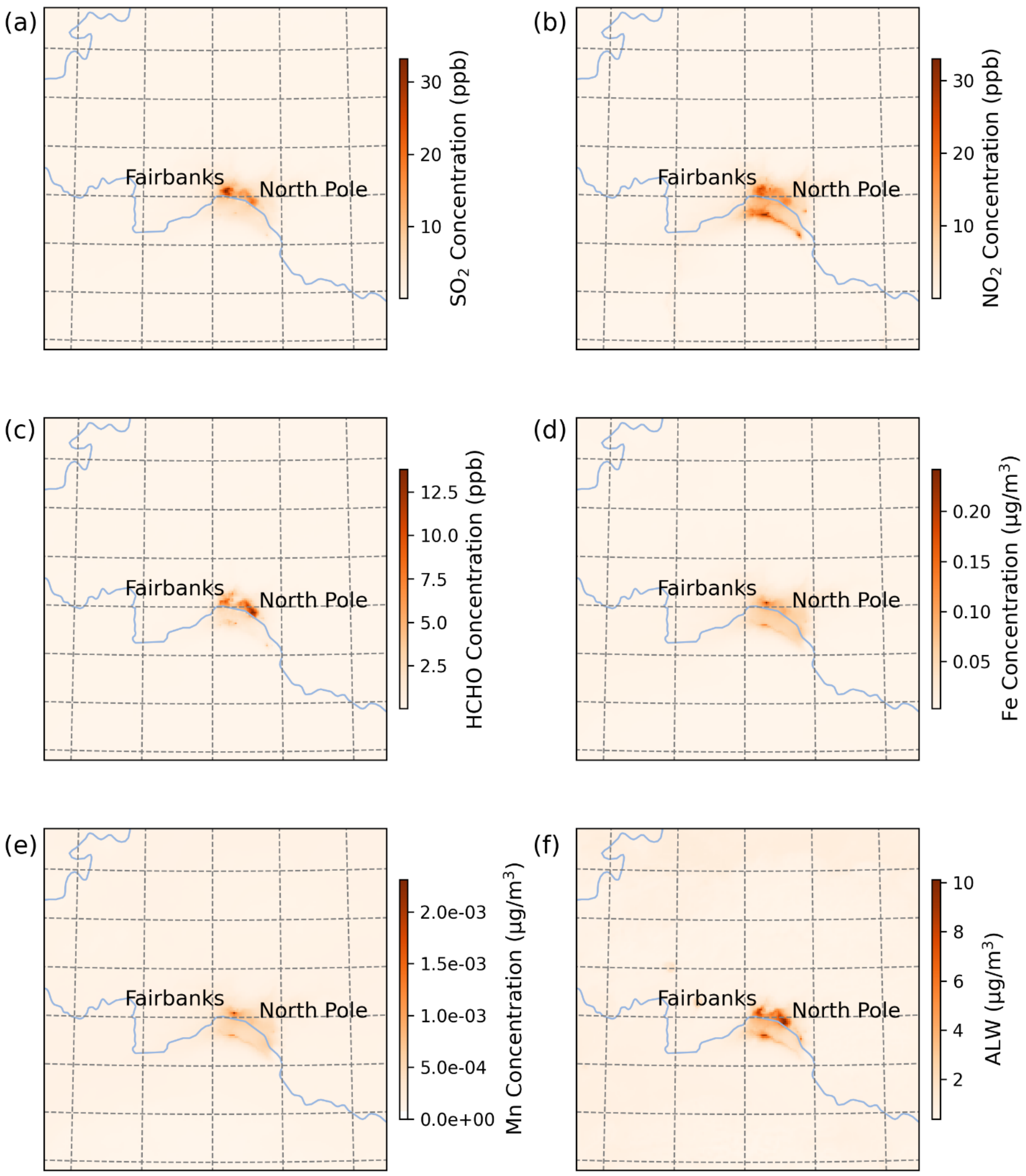

Figure S2. Episode averaged modelled ambient concentrations of PM2.5,sulf precursors in the Base_Het simulation for episode 1 (January 25th – February 11th ,2008) over the Fairbanks Domain.

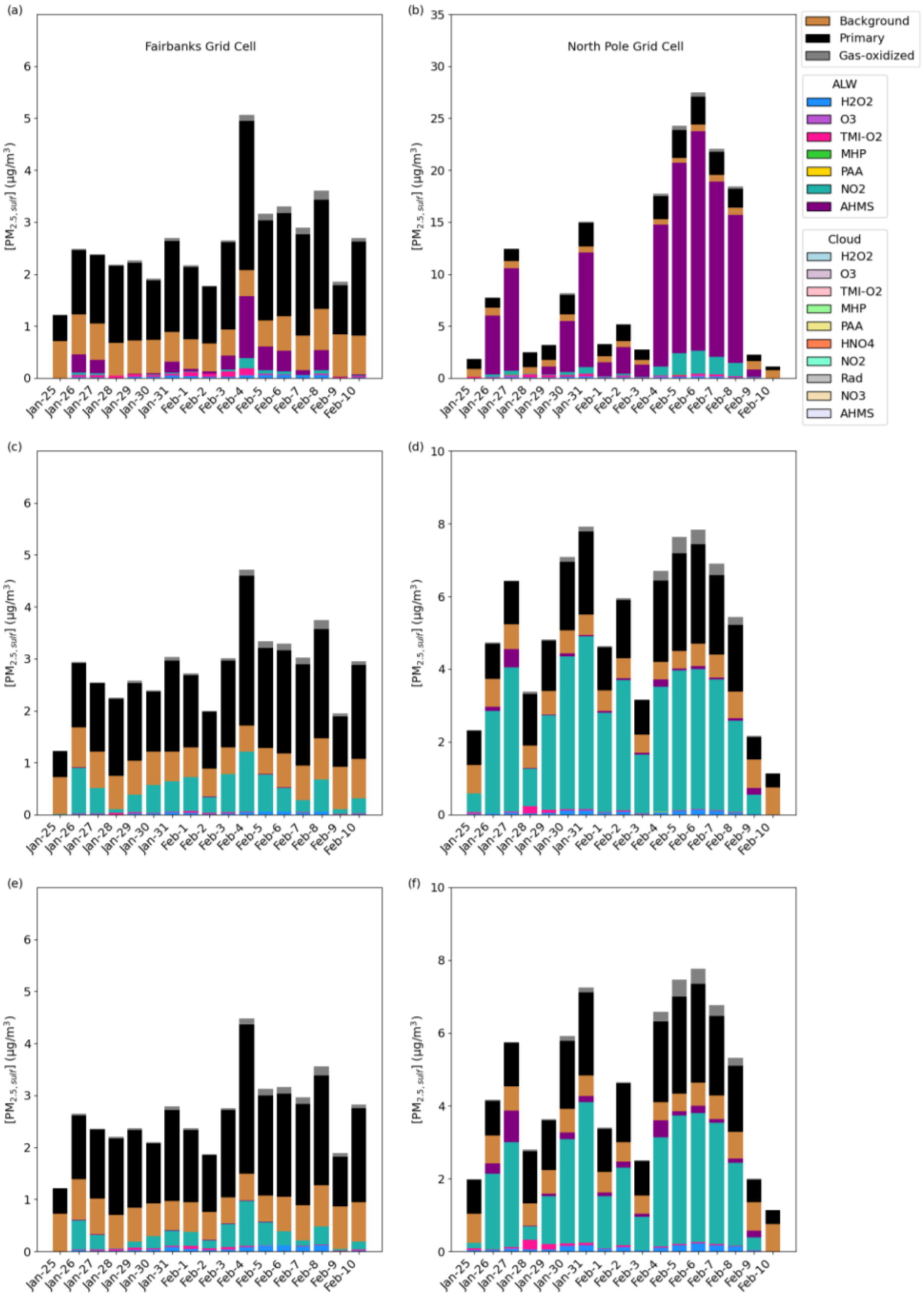

Figure S3. Average daily PM2.5,sulf concentrations in the TMI_sens (a & b), TMI_NO2_sens (c & d), and All_Ionic (e & f) simulations at Downtown Fairbanks (a, c & e) located at the State Office Building (64.84 N, -147.72 W; grid cell 108,93) and North Pole (b, d & f) located at (64.76 N, -147.34 W; grid cell 122, 86) for episode 1 speciated by source and/or formation pathway. Secondary aqueous formation of PM2.5,sulf is broken out into two categories: ALW and Cloud, where ALW pathways represent the heterogeneous sulfur chemistry added in this study.

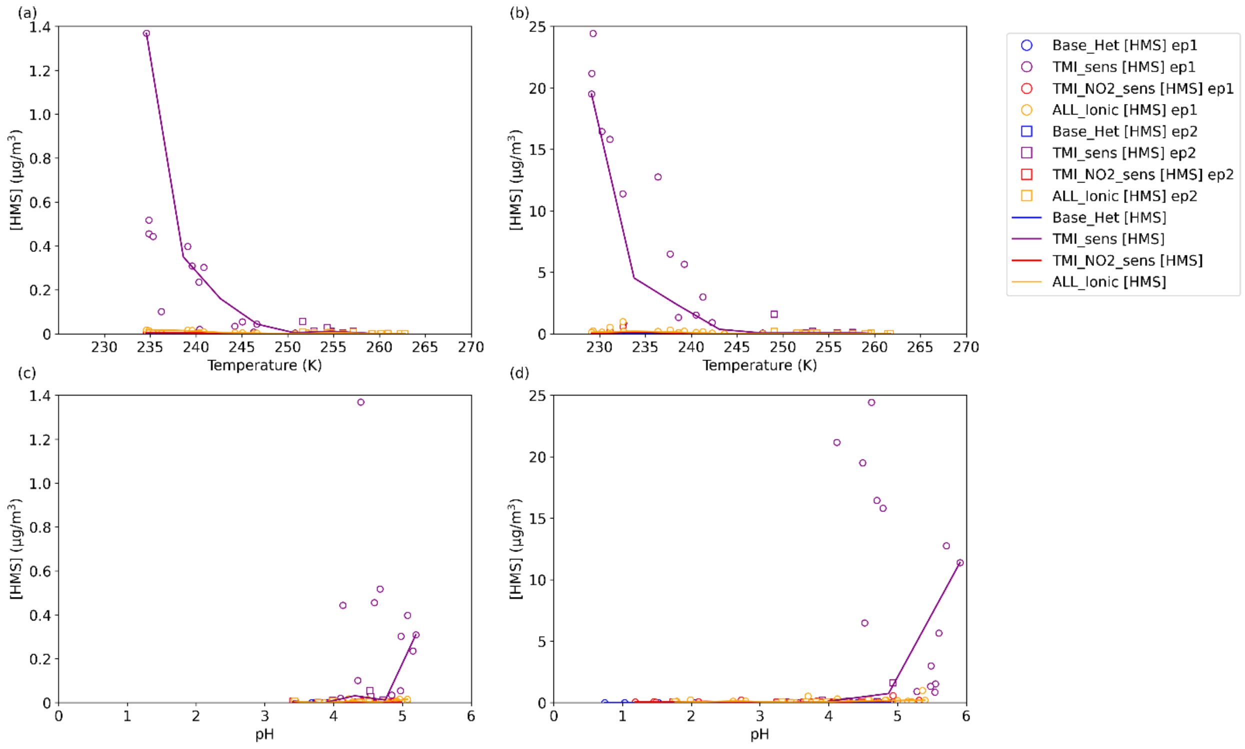

Figure S4.) Relationship between HMS formation and temperature (a & b) and HMS formation and pH (c & d) for the Fairbanks domain (both episodes) at the Downtown Fairbanks Grid Cell (a & c) and the North Pole Grid Cell (b & d). Scatters represent daily averages (17 points per model run for E1 and 12 points per model run for E2). Interpolated trend lines were calculated assuming a linear degree of B-spline at 8 intervals for HMS/Temperature relationships (a & b) and 5 intervals for HMS/pH relationships (c & d).

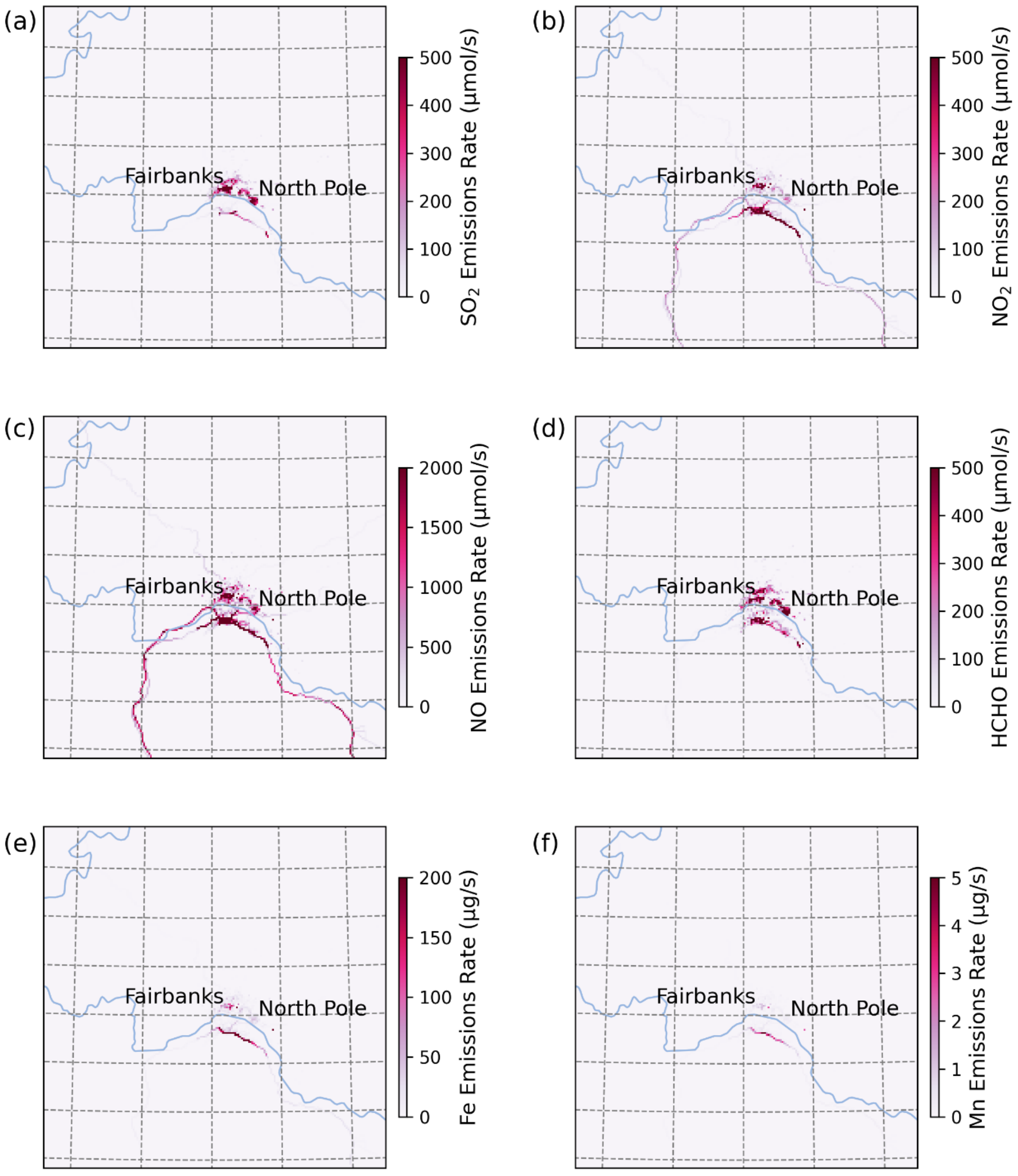

Figure S5. Average emissions of sulfur aerosol precursors for episode 2 (November 4th – November 17th, 2008)

35

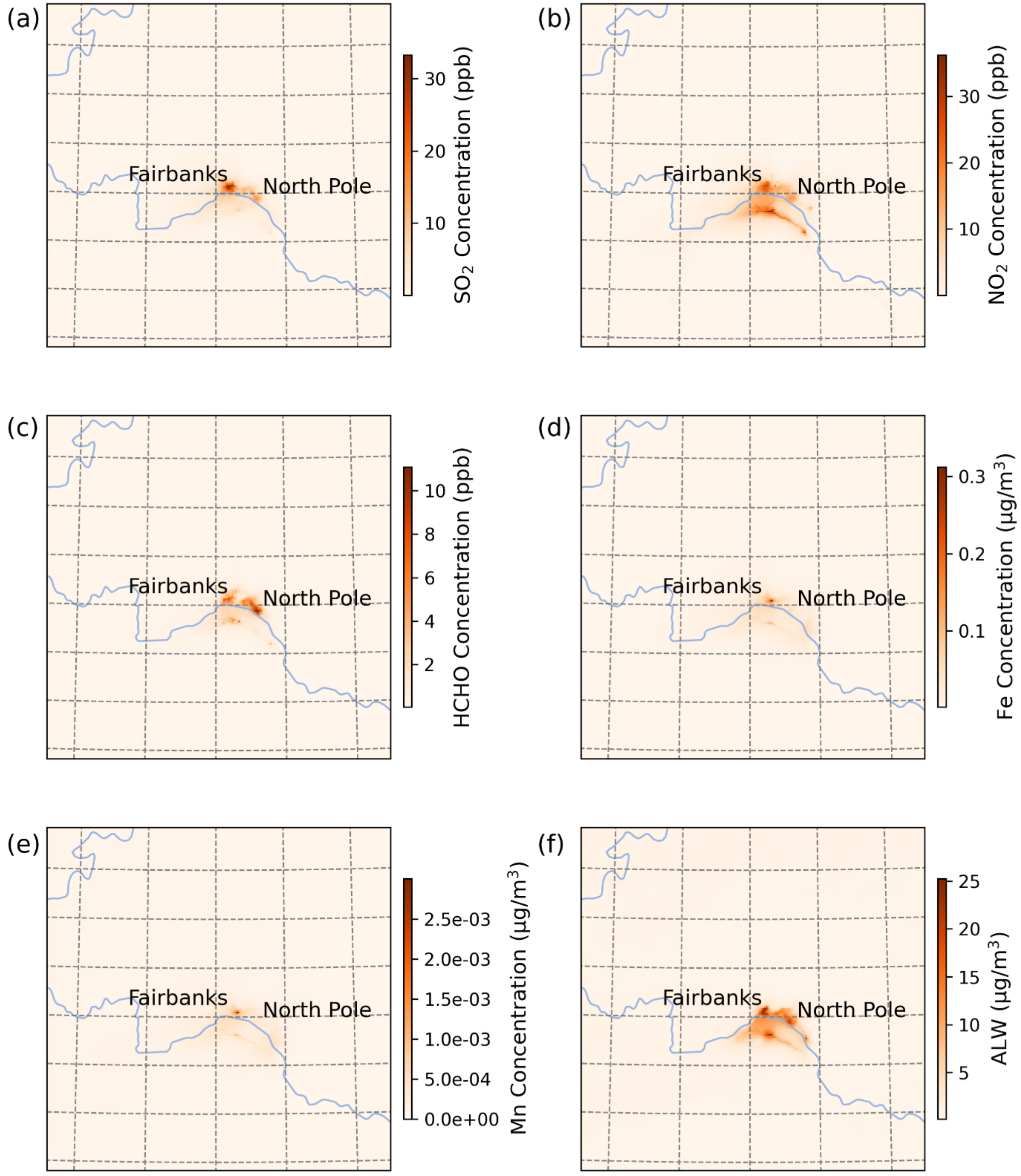

Figure S6. Episode averaged modelled ambient concentrations of PM2.5,sulf precursors in the Base_Het simulation for episode 2 (November 4th – 17th, 2008) over the Fairbanks Domain.

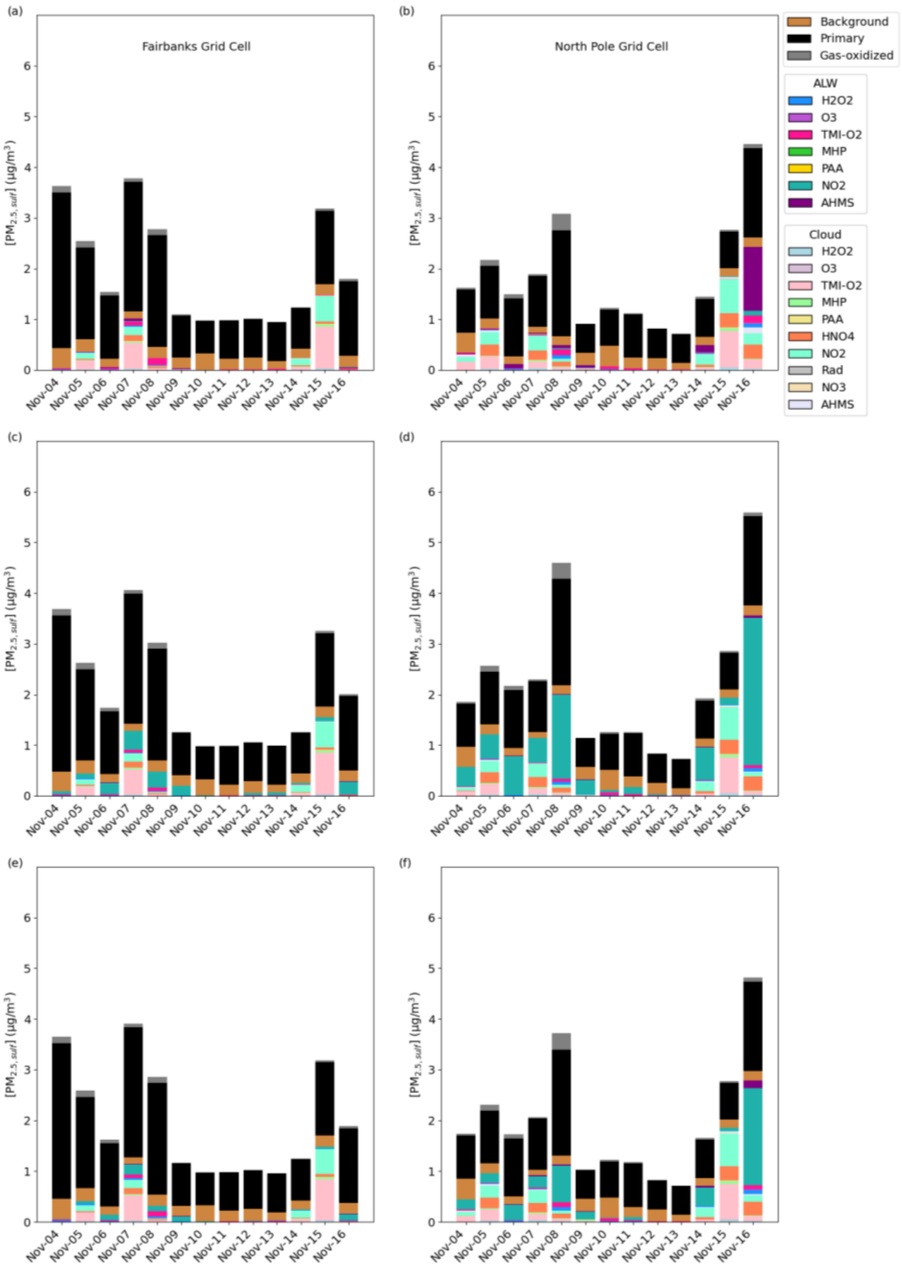

Figure S7. Average daily PM2.5,sulf concentrations in the TMI_sens (a & b), the TMI_NO2_sens (c & d), and the All_Ionic (e & f) simulations at Downtown Fairbanks (a, c & e) located at the State Office Building (64.84 N, -147.72 W; grid cell 108,93) and North Pole (b, d & f) located at (64.76 N, -147.34 W; grid cell 122, 86) for episode 2 speciated by source and/or formation pathway. Secondary aqueous formation of PM2.5,sulf is broken out into two categories: ALW and Cloud, where ALW pathways represent the heterogeneous sulfur chemistry added in this study.

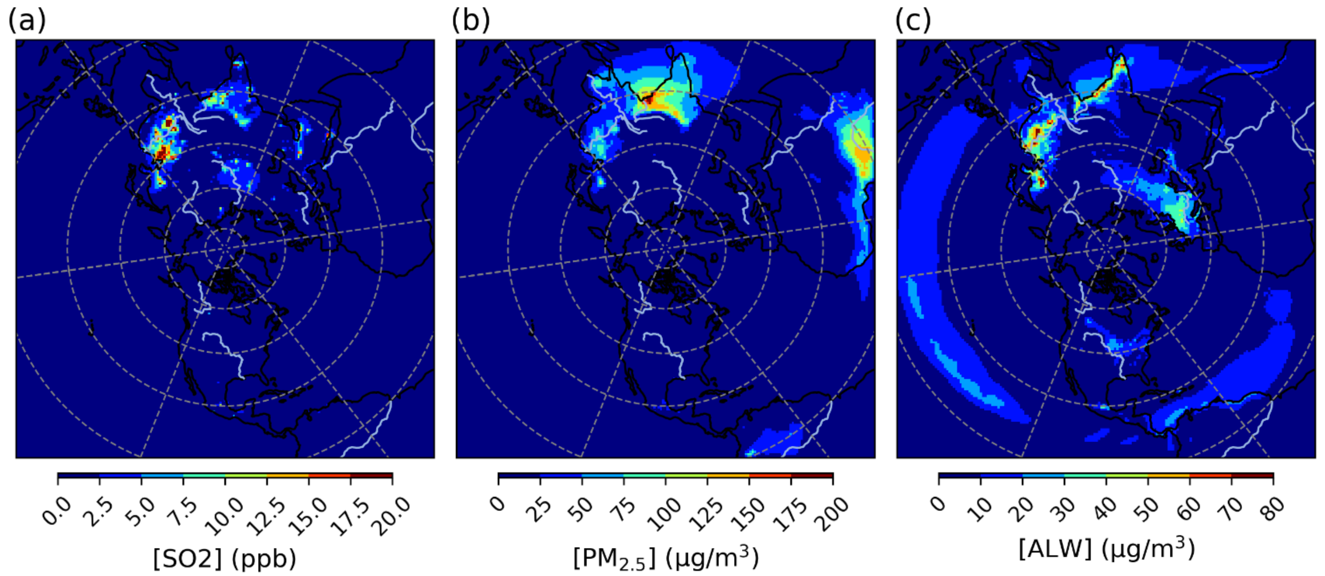

Figure S8. Episode Averaged SO_2 (a), $PM_{2.5}$ (b), and ALW (c) from Base HCMAQ for a wintertime episode (December 1st, 2015 – February 29th, 2016)

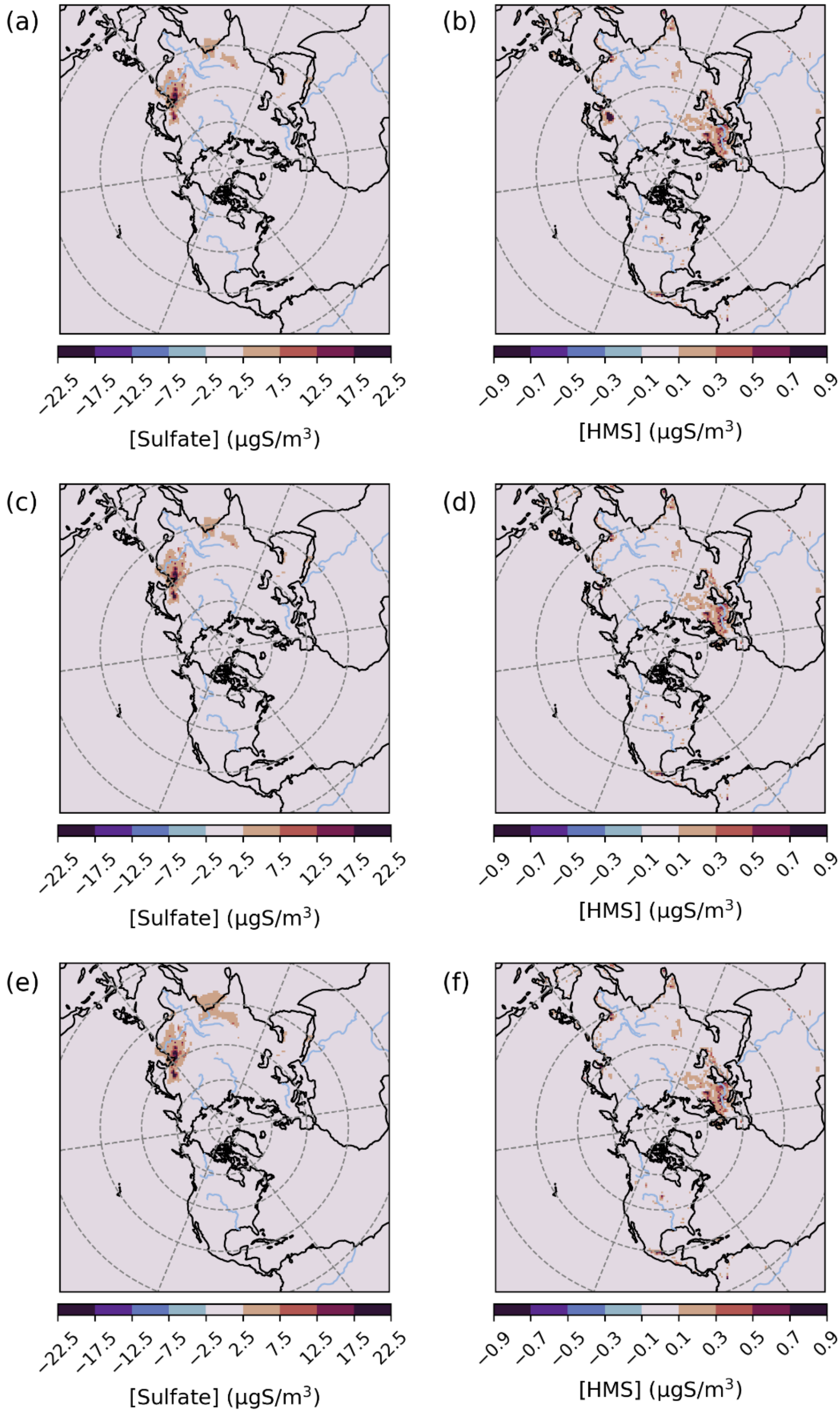
(a)
(b)
(c)
(d)
(e)
(f)
−22.5 −17.5 −12.5 −7.5 −2.5 2.5 7.5 12.5 17.5 22.5
[Sulfate] (μgS/m3)
−0.9 −0.7 −0.5 −0.3 −0.1 0.1 0.3 0.5 0.7 0.9
[HMS] (μgS/m3)

Figure S9. Maximum daily differences in sulfate and HMS for the TMI_sens (a&b), the TMI_NO2_sens (c & d) and the All_Ionic (e & f) model simulations over the HCMAQ N. Hemisphere domain for a wintertime episode (December 1st, 2015 – February 29th, 2016). Differences are cast in micrograms of sulfur per meter cubed to be consistent with measurement units from EMEP.

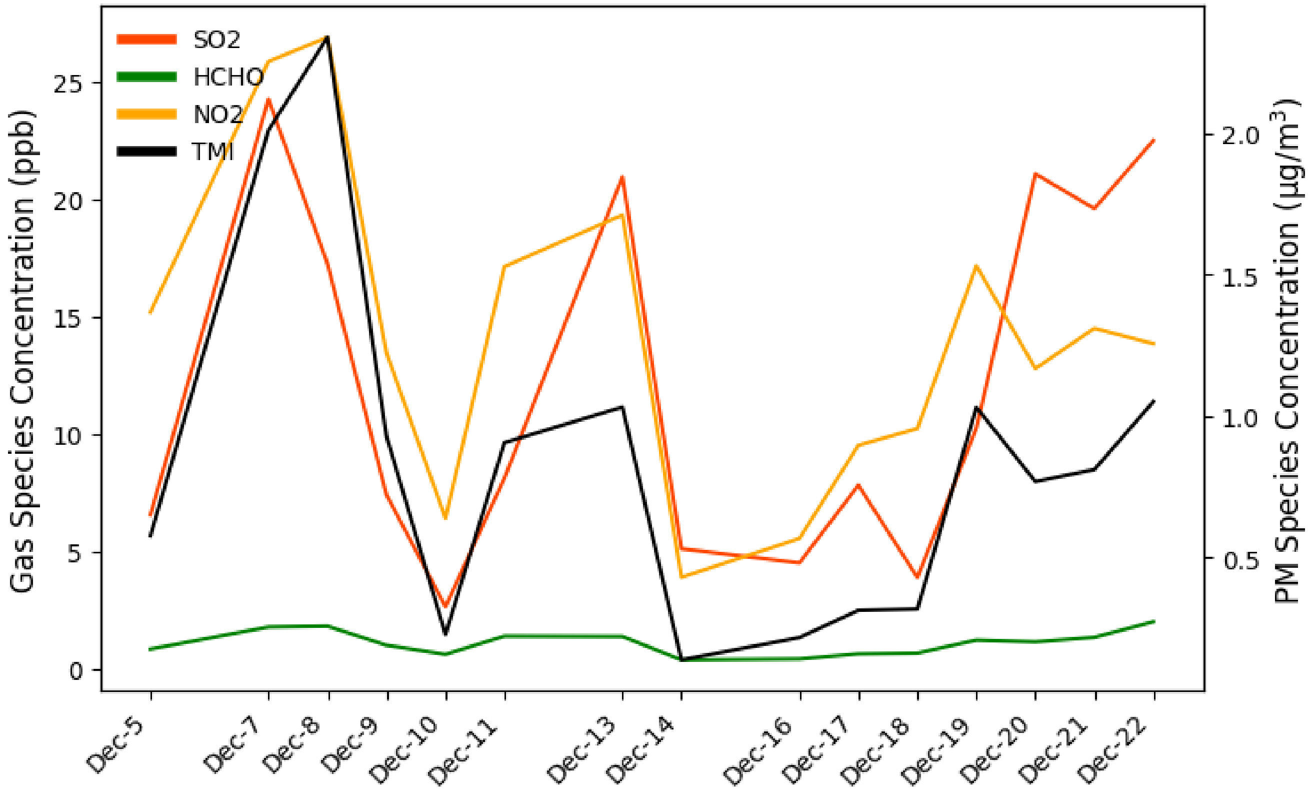

Figure S10. Time series of daily averaged secondary sulfate formation oxidant and catalyst concentrations for Base HCMAQ at a grid cell over Tsinghua University in Beijing, China from December 5th to December 22nd, 2015.

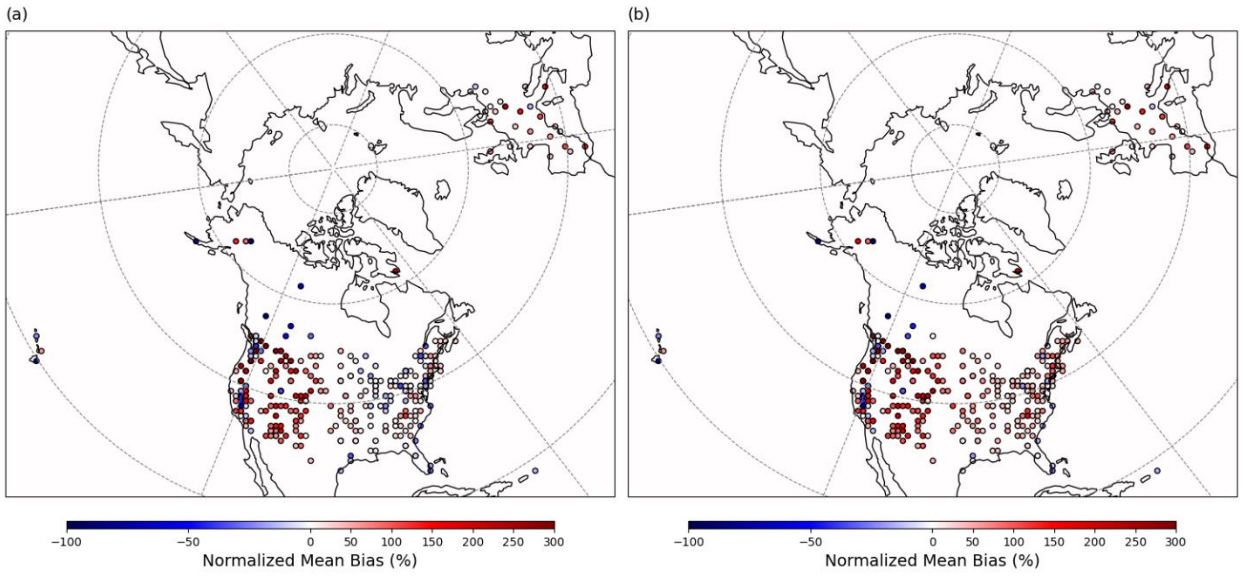

Figure S11. Normalized Mean Bias for $PM_{2.5,sulf}$ by monitor over the Hemispheric Domain for the winter season 2015-2016 for the Base (a) and Base_Het (b) model simulations.

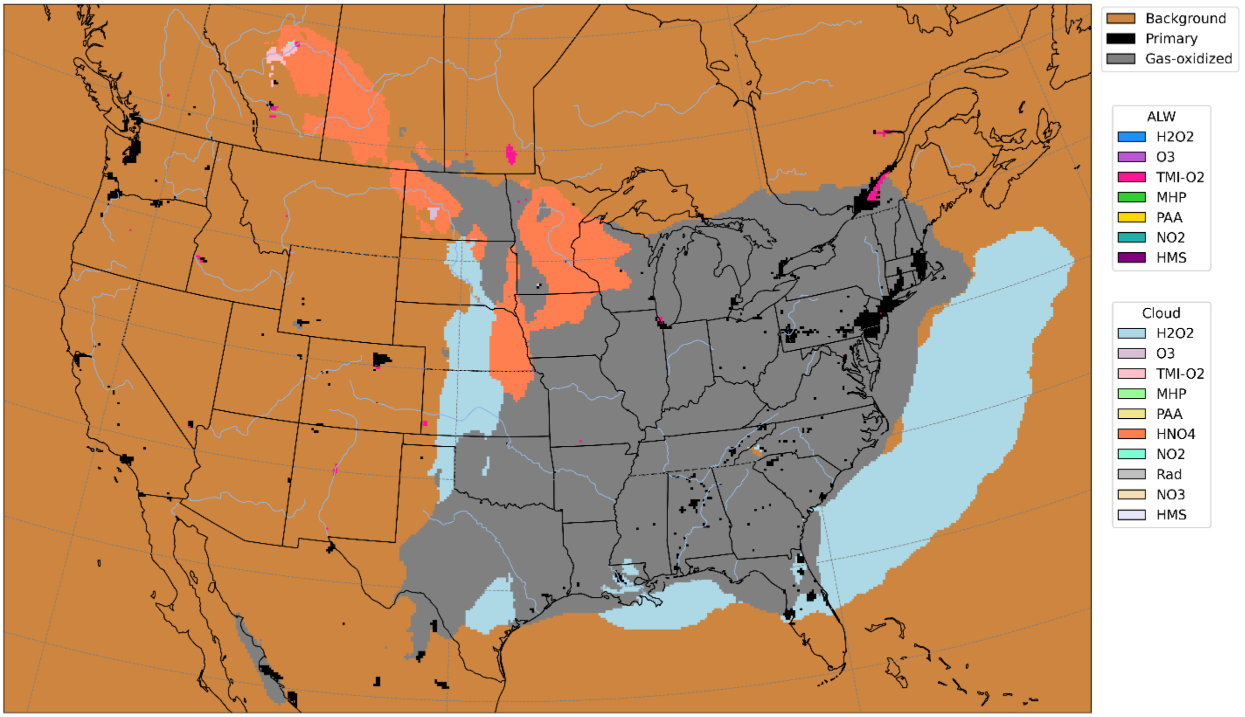

Figure S12. Leading contributor to PM2.5,sulf concentration for January 2016 over CONUS in the Base_Het model simulation.

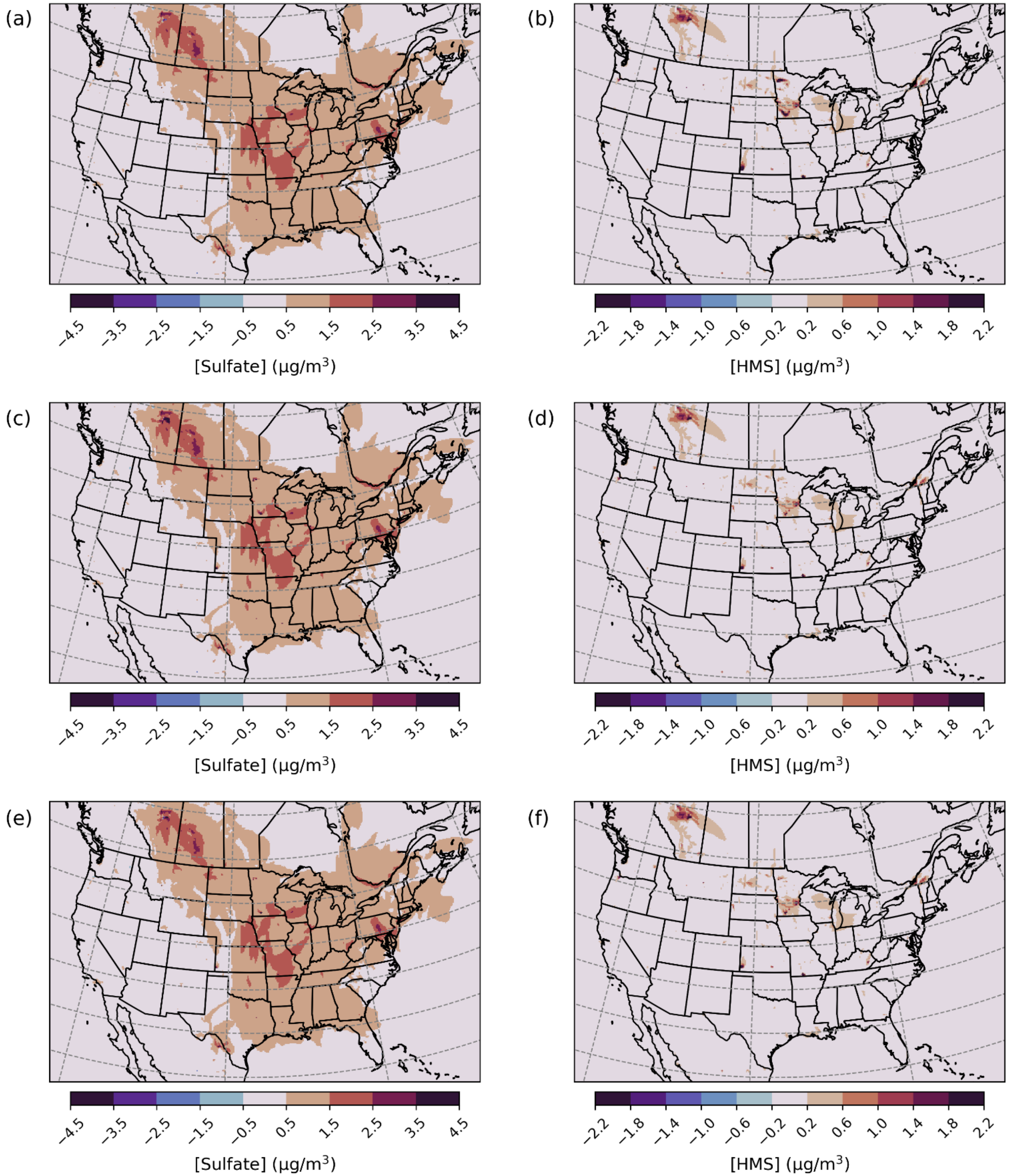
(a)
-4.5 -3.5 -2.5 -1.5 -0.5 0.5 1.5 2.5 3.5 4.5
[Sulfate] (μg/m3)
(b)
-2.2 -1.8 -1.4 -1.0 -0.6 -0.2 0.2 0.6 1.0 1.4 1.8 2.2
[HMS] (μg/m3)
(c)
-4.5 -3.5 -2.5 -1.5 -0.5 0.5 1.5 2.5 3.5 4.5
[Sulfate] (μg/m3)
(d)
-2.2 -1.8 -1.4 -1.0 -0.6 -0.2 0.2 0.6 1.0 1.4 1.8 2.2
[HMS] (μg/m3)
(e)
-4.5 -3.5 -2.5 -1.5 -0.5 0.5 1.5 2.5 3.5 4.5
[Sulfate] (μg/m3)
(f)
-2.2 -1.8 -1.4 -1.0 -0.6 -0.2 0.2 0.6 1.0 1.4 1.8 2.2
[HMS] (μg/m3)

Figure S13. Maximum daily differences in sulfate and HMS between Base CMAQ and the TMI_sens (a&b), the TMI_NO2_sens (c & d), and the All_Ionic (e & f) simulations for a wintertime episode (January 2016). HMS formation was not included in Base CMAQ, and HMS mass concentrations are multiplied by the ratio of sulfate to HMS molecular mass.

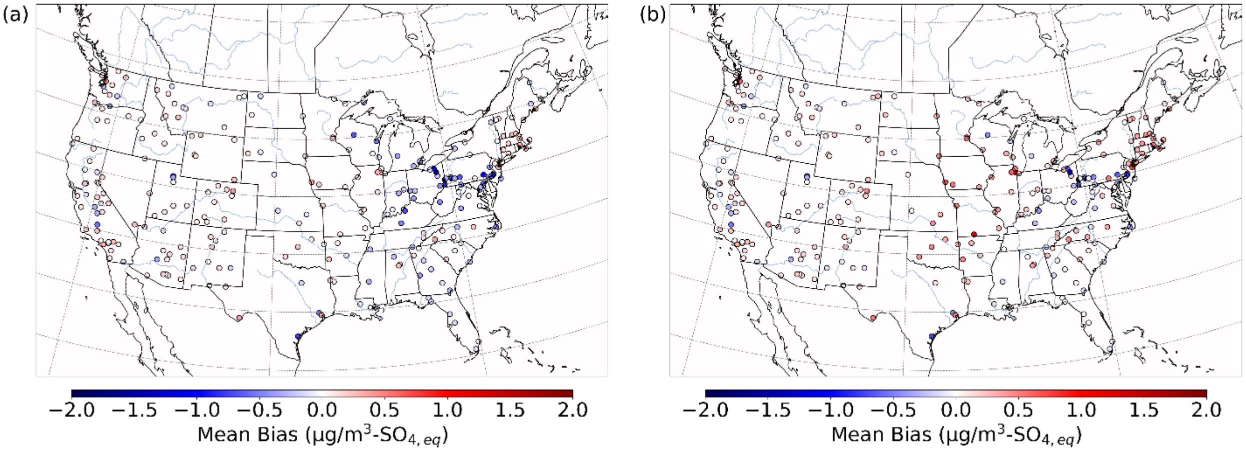

Figure S14. Mean bias calculated for modelled PM2.5,sulf in the Base (a) and Base_Het (b) simulation for a wintertime episode (January 2016) over the CONUS domain. Mean bias is cast in SO4 equivalence to account for and equivalate HMS contributions to sulfate measured mass in observations (HMS modelled concentrations are multiplied by the ratio of sulfate to HMS molecular mass).

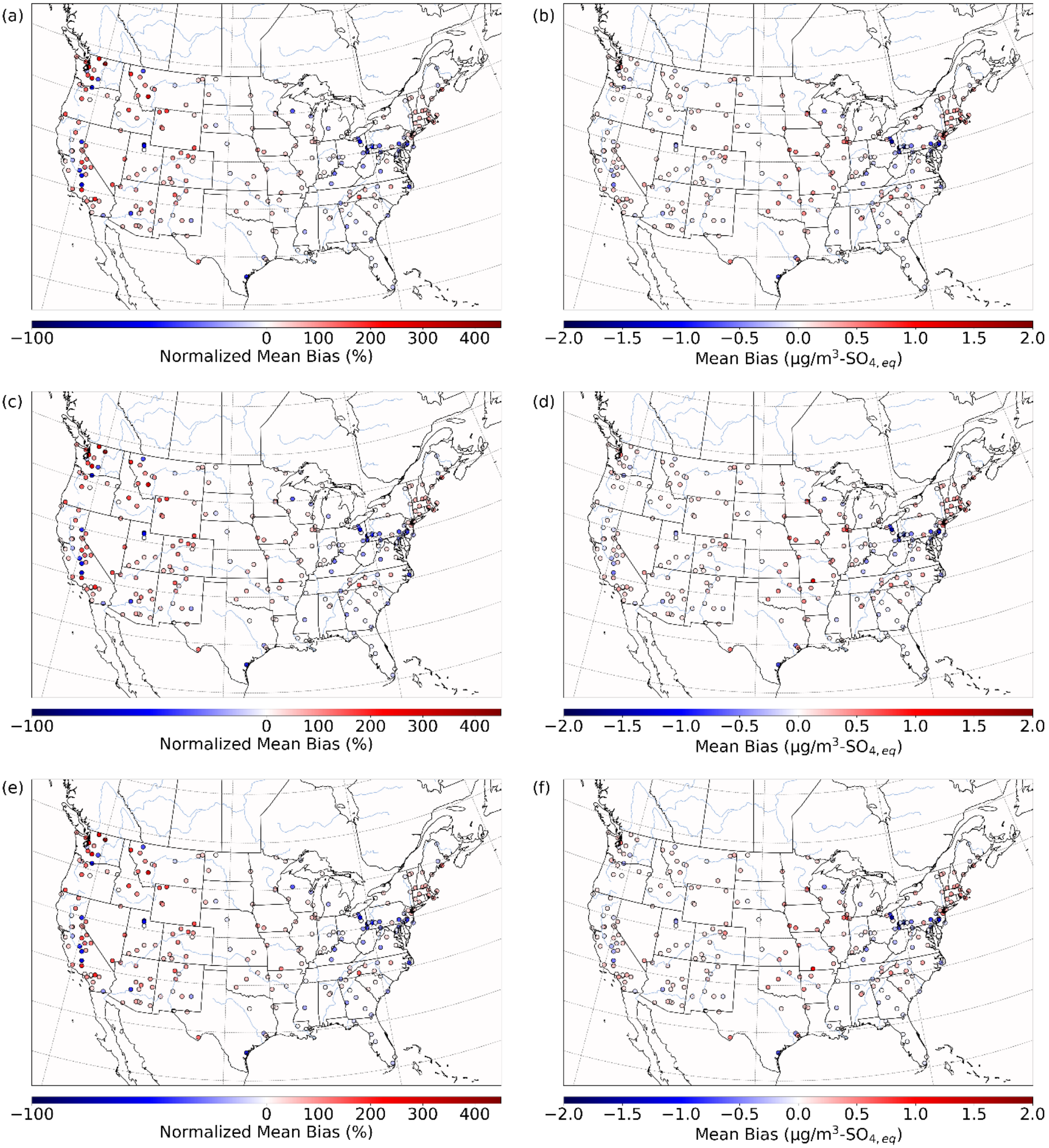

Figure S15. Normalized mean bias and mean bias in modelled PM2.5,sulf for a wintertime episode (January 2016) over the CONUS domain predicted by the TMI_sens (a & b), the TMI_NO2_sens (c & d), and the All_Ionic (e & f) simulations. Mean bias is cast in SO4 equivalence to account for and equivalate HMS contributions to sulfate measured mass in observations.

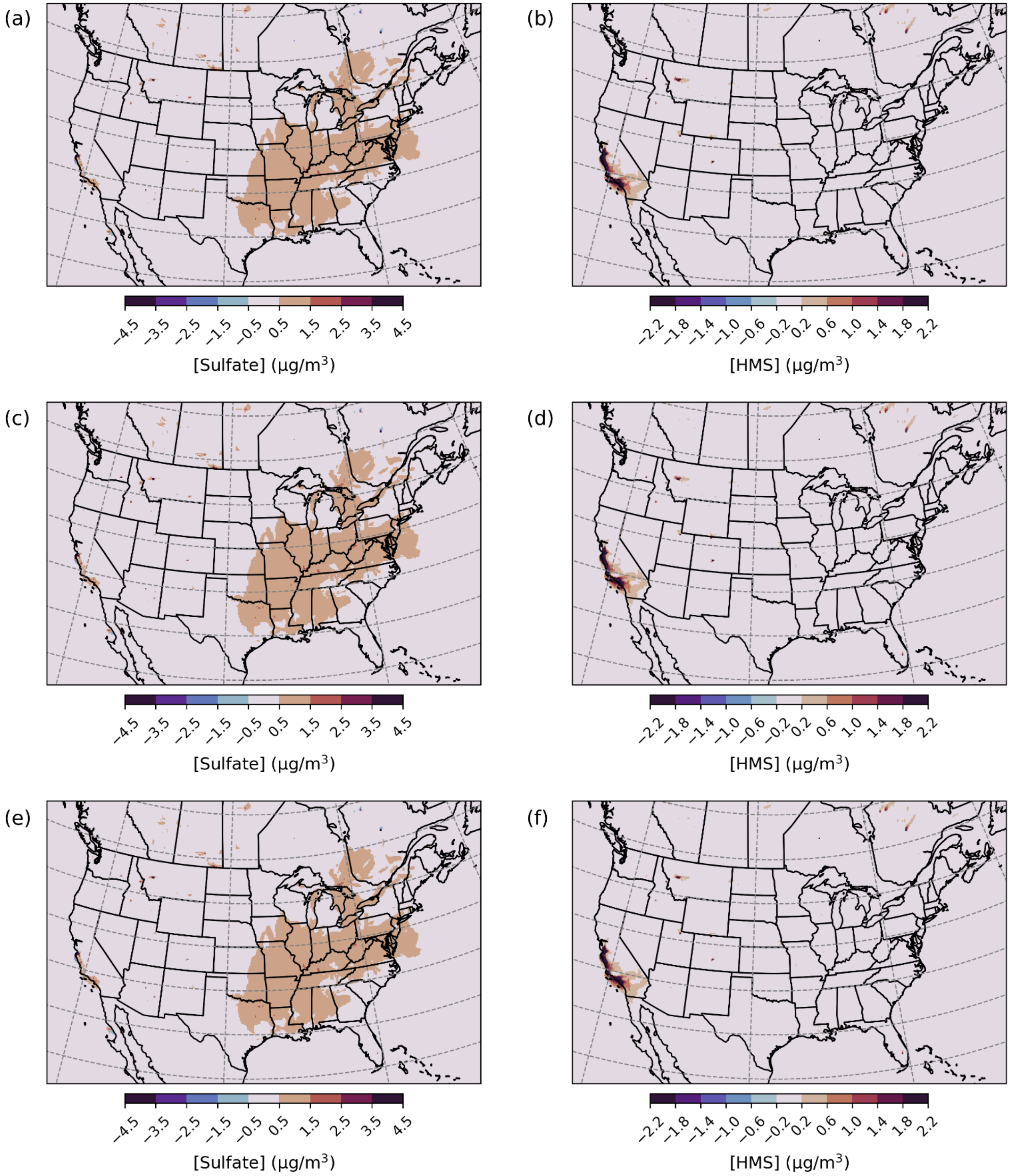
(a)
(b)
(c)
(d)
(e)
(f)
−4.5 −3.5 −2.5 −1.5 −0.5 0.5 1.5 2.5 3.5 4.5
[Sulfate] (μg/m3)
−2.2 −1.8 −1.4 −1.0 −0.6 −0.2 0.2 0.6 1.0 1.4 1.8 2.2
[HMS] (μg/m3)

Figure S16. Max daily differences in sulfate and HMS between Base CMAQ and the TMI_sens (a & b), the TMI_NO2_sens (c & d), and the All_Ionic (e & f) simulations for a summertime episode (July 2016).

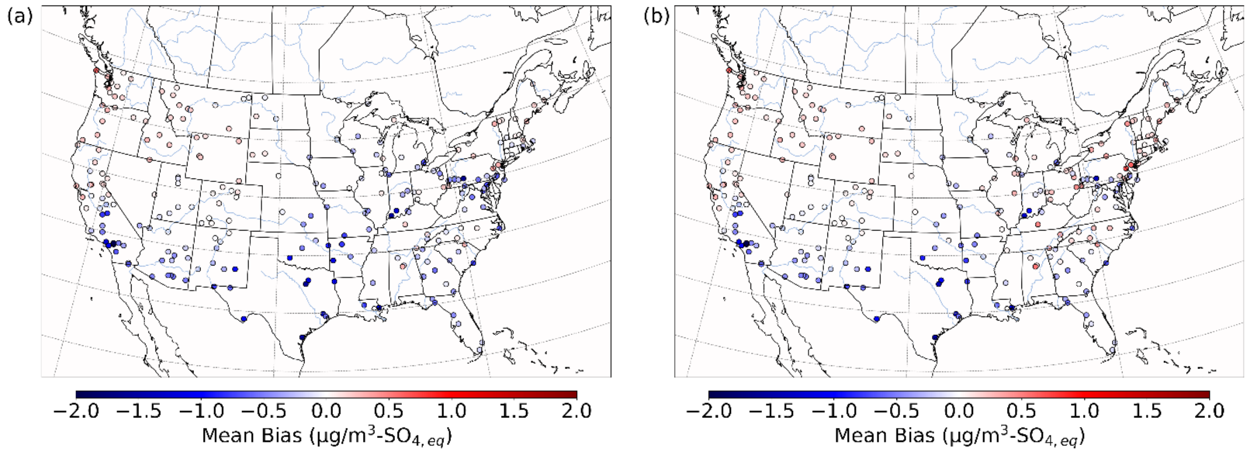

Figure S17. Mean bias calculated for modelled PM2.5,sulf in the Base (a) and Base_Het (b) simulation for a summertime episode (July 2016) over the CONUS domain. Mean bias is cast in SO4 equivalence to account for and equivalate HMS contributions to sulfate measured mass in observations.

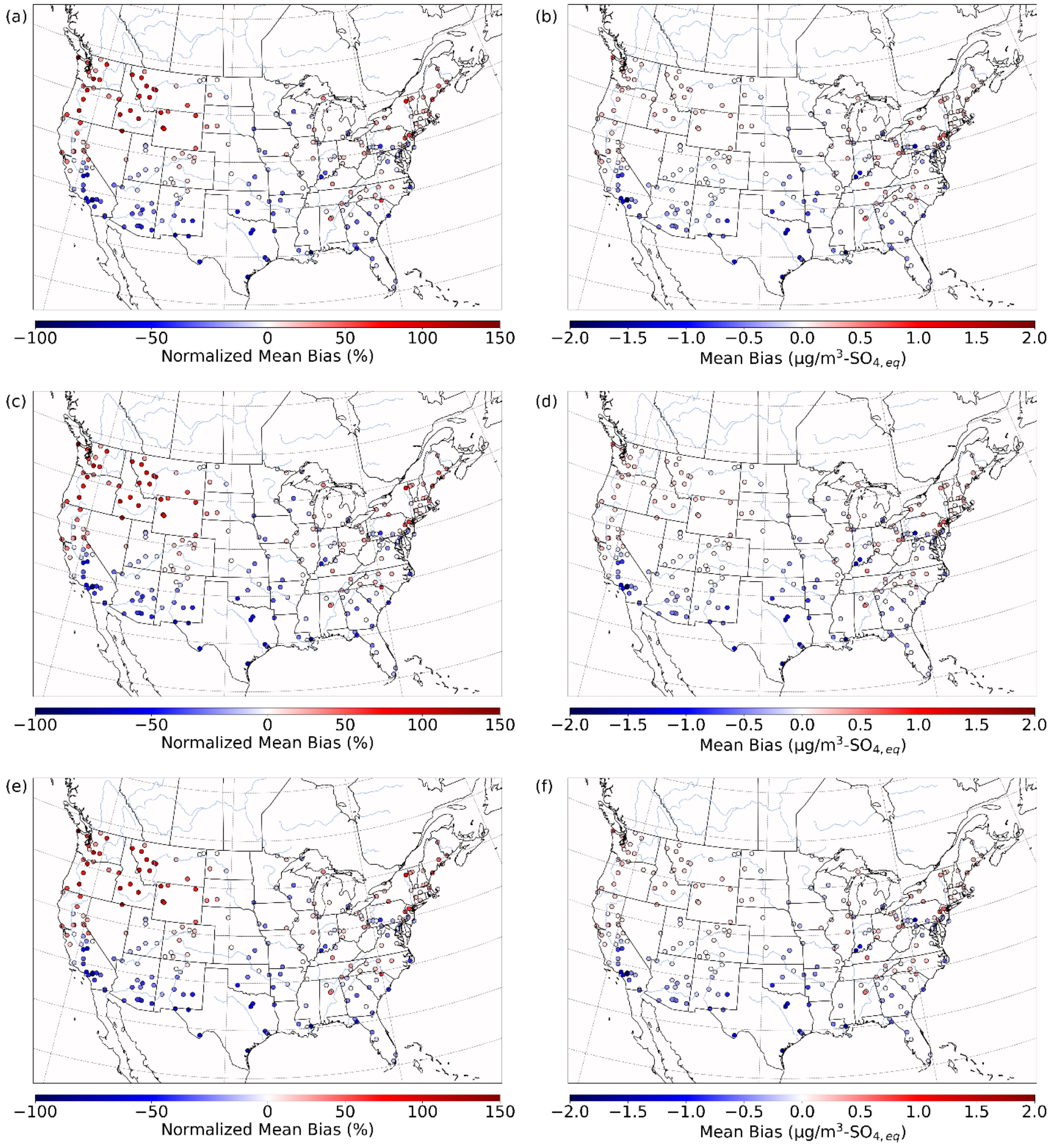

Figure S18. Normalized mean bias and mean bias in modelled PM2.5,sulf for a summertime episode (July 2016) over the CONUS domain predicted by the TMI_sens (a & b), the TMI_NO2_sens (c & d), and the All_Ionic (e & f) simulations.

Table S1. HCMAQ model performance metrics for all model runs by region, including, normalized mean bias (NMB), normalized mean error (NME), mean bias (MB), mean error (ME), root mean square error (RMSE), correlation coefficient (R2) and total observation-model match counts (n).

Region	Performance	Base	Base_Het	TMI_sens	TMI_NO2_sens	All_IONIC
Canada	NMB (%)	-14.23	7.38	4.31	4.51	4.60
	NME (%)	50.00	56.54	54.97	55.01	55.00
	MB (µg/m^3)	-0.04	0.02	0.01	0.01	0.01
	ME (µg/m^3)	0.15	0.17	0.17	0.17	0.17
	RMSE (µg/m^3)	0.29	0.31	0.30	0.30	0.30
	R^2	0.17	0.19	0.20	0.20	0.20
	n	421				
Europe	NMB (%)	25.35	26.93	26.75	26.80	26.15
	NME (%)	64.40	64.38	64.41	64.42	63.98
	MB (µg/m^3)	0.07	0.08	0.08	0.08	0.07
	ME (µg/m^3)	0.18	0.18	0.18	0.18	0.18
	RMSE (µg/m^3)	0.32	0.32	0.32	0.32	0.31
	R^2	0.21	0.24	0.23	0.23	0.24
	n	3402				
USA	NMB (%)	12.70	36.08	33.85	33.89	33.95
	NME (%)	61.19	73.04	71.97	71.97	71.90
	MB (µg/m^3)	0.03	0.08	0.08	0.08	0.08
	ME (µg/m^3)	0.14	0.17	0.16	0.16	0.16
	RMSE (µg/m^3)	0.24	0.27	0.27	0.27	0.27
	R^2	0.28	0.30	0.30	0.30	0.30
	n	7422				
